# Association of Urban Green Space With Mental Health and General Health Among Adults in Australia

**DOI:** 10.1001/jamanetworkopen.2019.8209

**Published:** 2019-07-26

**Authors:** Thomas Astell-Burt, Xiaoqi Feng

**Affiliations:** 1 Population Wellbeing and Environment Research Lab (PowerLab), School of Health and Society, Faculty of Social Sciences, University of Wollongong, Wollongong, New South Wales, Australia

## Abstract

**Importance:**

Recent studies indicate that living near more green space may support mental and general health and may also prevent depression. However, most studies are cross-sectional, and few have considered whether some types of green space matter more for mental health.

**Objective:**

To assess whether total green space or specific types of green space are associated with better mental health.

**Design, Setting, and Participants:**

This cohort study included a residentially stable, city-dwelling sample of 46 786 participants from Sydney, Wollongong, and Newcastle, Australia, in the baseline of the Sax Institute’s 45 and Up Study (data collected from January 1, 2006, to December 31, 2009). Follow-up was conducted from January 1, 2012, to December 31, 2015. Analyses were conducted in January 2019.

**Exposures:**

Percentage of total green space, tree canopy, grass, and other low-lying vegetation measured within 1.6-km (1-mile) road network distance buffers around residential addresses at baseline.

**Main Outcomes and Measures:**

Three outcome variables were examined at baseline (prevalence) and follow-up (incidence without baseline affirmatives): (1) risk of psychological distress (10-item Kessler Psychological Distress Scale), (2) self-reported physician-diagnosed depression or anxiety, and (3) fair to poor self-rated general health.

**Results:**

This study included 46 786 participants (mean [SD] age, 61.0 [10.2] years; 25 171 [53.8%] female). At baseline, 5.1% of 37 775 reported a high risk of psychological distress, 16.0% of 46 786 reported depression or anxiety, and 9.0% of 45 577 reported fair to poor self-rated health. An additional 3.3% of 32 991 experienced psychological distress incidence, 7.5% of 39 277 experienced depression or anxiety incidence, and 7.3% of 40 741 experienced fair to poor self-rated health incidence by follow-up (mean [SD] of 6.2 [1.62] years later). Odds ratios (ORs) adjusted for age, sex, income, economic status, couple status, and educational level indicated that exposures of 30% or more total green space (OR, 0.46; 95% CI, 0.29-0.69) and tree canopy specifically (OR, 0.69; 95% CI, 0.54-0.88) were associated with lower incidence of psychological distress. Exposure to tree canopy of 30% or more, compared with 0% to 9%, was also associated with lower incidence of fair to poor general health (OR, 0.67; 95% CI, 0.57-0.80). Exposure to grass of 30% or more, compared with 0% to 4%, was associated with higher odds of incident fair to poor general health (OR, 1.47; 95% CI, 1.12-1.91) and prevalent psychological distress (OR, 1.71; 95% CI, 1.25-2.28). Exposure to low-lying vegetation was not consistently associated with any outcome. No green space indicator was associated with prevalent or incident depression or anxiety.

**Conclusions and Relevance:**

Protection and restoration of urban tree canopy specifically, rather than any urban greening, may be a good option for promotion of community mental health.

## Key Points

**Question:**

What type of green space is associated with better mental health?

**Findings:**

In this cohort study of 46 786 adults older than 45 years, exposure to 30% or more tree canopy compared with 0% to 9% tree canopy was associated with 31% lower odds of incident psychological distress, whereas exposure to 30% or more grass was associated with 71% higher odds of prevalent psychological distress after adjusting for age, sex, income, economic status, couple status, and educational level. Similar results were found for self-rated fair to poor general health but not physician-diagnosed depression or anxiety.

**Meaning:**

Investments specifically in tree canopy may provide more support for mental health.

## Teaser

This cohort study assesses whether urban green space, tree canopy, grass, and other low-lying vegetation are associated with better mental health among Australian adults.

## Introduction

The foreword by Margaret Chan, MD, to the World Health Organization’s Mental Health Action Plan 2013-2020 stated that “good mental health enables people to realize their potential, cope with the normal stresses of life, work productively, and contribute to their communities.”^1^^(p 5)^ The action plan advocated for a multisectoral approach toward prevention of and enhanced recovery from mental ill-health, promotion of mental well-being, and reduction in disability and mortality among people living with mental disorders. Modifiable environmental factors to which people are exposed are potentially key upstream levers for promotion of community mental health.^2^ One such factor is green space.^3^

Recently published findings from a randomized clinical trial suggested that the greening of vacant lots can result in meaningful reductions in psychological distress.^4^ Urban greening within cities could promote mental health through various concomitant and potentially synergistic domain pathways now increasingly referred to as (1) restoring capacities, (2) building capacities, and (3) reducing harm.^5^ Simply being in, nearby, or with a view of green space may help to build capacities for better mental health, contribute to restoration of depleted cognitive capacities, enhance recovery from periods of psychosocial stress, and even increase optimism.^6,7,8,9,10,11,12^ Amplification of these mental health benefits may occur in part as a result of social and physical recreation within green spaces.^13,14,15^ Nearby green space can also contribute natural, biodiverse soundscapes that soothe,^16^ dampen chronic noise,^17^ and potentially even disrupt the effect of socioeconomic disadvantage on mental ill-health.^18^

The presence of a particular type of green space may be an important condition for supporting several of these domain pathways. For example, some work in Australia suggests that humans prefer to seek green spaces with higher density and moderate vegetation complexity reflective of tree canopy rather than relatively simple open spaces more akin to large areas of grass.^19^ Other work has similarly found differences in preferences between green space types with respect to restoration via being away and fascination.^20^ Plain, flat grassy areas may not be particularly attractive for walking, which is an important form of social and physical recreation for older adults.^21^ Some work has found that tree canopy density specifically, rather than the presence of grass or parks in general, is associated with higher levels of social capital.^22^ Entwined with this is a potential indirect pathway via a third variable, such as the mental health impacts of heat islands,^23^ with tree canopy likely to be a better strategy for mitigating heat in cities than low-lying vegetation.^24^

However, most epidemiologic studies^25,26^ of green space and mental health have been of cross-sectional design, and reverse causation is a major concern.^25^ Furthermore, most studies,^25,26^ including many of longitudinal design, have been restricted to the conclusion that better mental health is associated with more greenery because there have been only a few explicit analyses^27,28^ of different types of green space exposure. Few studies have asked whether all types of green spaces confer the same potential health benefit. A cross-sectional study^28^ in the United States suggested the presence of forest and urban green space may support fewer days of mental health issues for city dwellers. Another cross-sectional study^27^ in the United Kingdom observed higher prevalence of self-rated good health in areas with green space types described as broadleaf woodland, arable and horticulture, and improved grassland but no associations with coniferous woodland, seminatural grassland, or mountain, heath, or bog.

To increase the utility of the mental health–related evidence being produced for decision-makers in urban planning and landscape architecture, longitudinal studies capable of distinguishing between different types of urban green space are warranted to give more specific guidelines on what could be achieved and how. In this longitudinal study, we addressed this gap in knowledge by asking whether all types of green spaces are associated with the same potential mental health benefit.

## Methods

In this cohort study, assessment of green space indicators with respect to prevalence and incidence (without baseline affirmatives) of 3 different mental health–related outcomes were examined in 46 786 participants who did not change residence between baseline (January 1, 2006, to December 31, 2009) and follow-up (January 1, 2012, to December 31, 2015). These cohort data were extracted in January 2019 from the Sax Institute’s 45 and Up Study^29^ and included participants living in the cities of Sydney, Wollongong, or Newcastle, Australia. The Department of Human Services (formerly Medicare Australia) enrollment database was originally used to randomly sample and then recruit participants at baseline using a postal survey, which provided near-complete coverage of the population of Australia. All participants in the 45 and Up Study gave written informed consent for their data to be used for research purposes. All data were deidentified. Ethics approval for the 45 and Up Study was provided by The University of New South Wales Human Research Ethics Committee. Ethics approval for this study was provided by the University of Wollongong Human Research Ethics Committee. This study followed the Strengthening the Reporting of Observational Studies in Epidemiology (STROBE) reporting guideline.

 The Australian Bureau of Statistics (ABS) Urban Centre and Locality boundaries were used to define the metropolitan areas of Sydney, Newcastle, and Wollongong. Sydney is the most populous city in Australia, with 4 321 535 people, and the capital of the state of New South Wales, the most populous state in the country, with 7 480 228 people according to the 2016 Australian Census. Newcastle and Wollongong are 2 other large cities in New South Wales, with 322 278 people in Newcastle and 261 896 people in Wollongong. The sample was restricted to only those participants living within those cities who resided in the same neighborhoods (proxied by statistical area 2 [SA 2]) at baseline and follow-up (eFigure in the Supplement).

### Outcomes

Three outcome variables were examined at baseline (prevalence) and follow-up (incidence, without baseline affirmatives): (1) risk of psychological distress, (2) self-reported physician-diagnosed depression or anxiety, and (3) self-rated fair to poor general health. The 10-item Kessler Psychological Distress Scale^30^ was used to measure the risk of psychological distress. This involved summing responses to 10 questions: “During the past 4 weeks, about how often did you feel tired out for no good reason, nervous, so nervous that nothing could calm you down, hopeless, restless or fidgety, so restless that you could not sit still, depressed, that everything was an effort, so sad that nothing could cheer you up, and worthless?” Responses to each of these 10 questions included none of the time (1 point), a little of the time (2 points), some of the time (3 points), most of the time (4 points), or all of the time (5 points). Total scores of 22 or higher were considered to be indicative of a high risk of psychological distress, in line with previous literature.^30^

Self-reported physician-diagnosed depression or anxiety was measured using affirmative responses to either of 2 questions: “Has a doctor ever told you that you have depression or anxiety?” and “In the last month have you been treated for depression or anxiety?” Self-rated fair to poor general health was assessed at baseline and follow-up with the question: “In general, how would you rate your overall health? Excellent, very good, good, fair, or poor?” Responses for fair and poor were aggregated (score of 1) and contrasted with the similarly aggregated alternatives (score of 0) as a dichotomous variable.

Prevalence of each of these outcomes was defined as affirmative responses at baseline contrasted with nonaffirmative responses. Participants with missing outcome data at baseline were excluded from analyses. Incidence was examined using affirmative responses at follow-up among a sample of participants restricted to those with nonmissing nonaffirmative outcomes at baseline.

### Green Space Data

The residential location of each participant in the 45 and Up Study was measured by the centroid of the ABS mesh block in which they lived at the time of recruitment into the baseline survey. Mesh blocks are the smallest geographical unit provided by the ABS, containing just 30 to 60 dwellings each. Road network distance–based buffers of 1.6-km (1-mile) radius were calculated in ArcGIS Pro (Esri) around each mesh block centroid and used to calculate the percentage of nearby land use corresponding with multiple indicators of green space provision.

Raster land-use data (Geovision) was licensed from Pitney Bowes Ltd for 2016. This 2-m raster was captured using machine learning and image classification processes across satellite imagery (8-band multispectral imagery captured by DigitalGlobe’s Worldview 3 satellite) to classify the surface into descriptive classes. A geographic information system was used to calculate percentages of total green space and separate green space types, including tree canopy, grass, or other low-lying vegetation across metropolitan areas of Sydney, Newcastle, and Wollongong. Trees included deciduous and evergreen woody vegetation, whereas grass included herbaceous areas. Other low-lying vegetation referred to other vegetative material not included within the grass or tree classes (eg, scrub). It was not possible to differentiate between green space types that overlapped using these data (eg, tree canopy that overlaps low-lying vegetation and/or grass). As such, the indicators of grass and low-lying vegetation are underestimates because they refer only to those provisions that were not beneath tree canopy.

The total green space and grass percentages were expressed a priori in the following intervals: 0% to 4%, 5% to 9%, 10% to 19%, 20% to 29%, and 30% or more. For tree canopy (0%-9%, 10%-19%, 20%-29%, and ≥30%) and low-lying vegetation (0%-4%, 5%-9%, and ≥10%), some intervals were aggregated because of small numbers.

### Confounding

Self-rated health, depression, anxiety, and risk of psychological distress have been previously shown to be associated with green space in some cross-sectional and longitudinal studies.^25,26^ A range of socioeconomic and demographic factors are likely to confound these associations by contributing to mental health outcomes and to neighborhood selection. Previous research suggests that these factors are likely to include personal socioeconomic circumstances, such as how much money people have, whether they are employed, and their level of education, and other factors, such as age, sex, and relationship status.^2^ Accordingly, in this study, we adjusted for baseline measures of age, sex, annual household income, economic status (eg, employed, retired, or unemployed), highest educational qualification, and couple status.

### Statistical Analysis

The patterning of missing, prevalent, and incident outcome data was assessed with respect to each of the land-use exposures and markers of potential confounding using cross-tabulations, percentages, and χ^2^ values with *P* < .05 considered to be statistically significant. Multilevel logistic regressions fitted with the Markov Chain Monte Carlo method in MLwIN^31^ were used to test associations between each of the above-mentioned outcomes and green space variables before and after adjusting for markers of confounding. Output variables from the fixed part of the models were odds ratios (ORs) and 95% CIs.

The longitudinal multilevel models had 4 levels, with person at level 1 and SAs 2, 3, and 4 at levels 2, 3, and 4, respectively. SA2s are medium-sized geographical boundaries that comprise a mean of 10 000 residents and are suggested by the ABS to represent spaces in which a community comes together socially and economically. SA3s are aggregations of SA2s and represent populations of 30 000 to 130 000 people in local government areas (council areas) and major transportation and commercial hubs. SA4s are aggregations of SA3s and reflect labor markets with a mean of 300 000 to 500 000 residents. All 3 geographic areas were assessed simultaneously within the multilevel models to disentangle spatial patterns of each outcome manifesting across each city with respect to local communities, councils, transportation and commercial areas, and broader labor markets.

## Results

This study included 46 786 participants (mean [SD] age, 61.0 [10.2] years; 25 171 [53.8%] female). Of these participants, 9011 (19.3%) were missing data for psychological distress and 1209 (2.6%) were missing data for self-rated general health (eTables 1-3 in the Supplement). No data were missing for the depression or anxiety outcome at baseline. Among participants with nonmissing nonaffirmative responses at baseline, 2845 of 35 836 (7.9%) were missing psychological distress data, 8 of 39 277 (0.02%) were missing depression or anxiety data, and 753 of 41 494 (1.8%) were missing general health data. Missing data for psychological distress and general health were more common among women, older people, people with lower incomes, people with lower educational qualifications, and those who were not employed or living in a couple. Missing psychological distress and self-rated general health data were also more common for people with less green space overall within 1.6 km, less tree canopy, and more grass (for psychological distress only). No substantive patterns were discernible for missing depression or anxiety outcome data, and no differences were found with regard to low-lying vegetation provision.

In the baseline sample of 46 786 participants, mean (SD) follow-up was 6.2 (1.62) years (range, 2.25-10.73 years). A total of 17 611 (37.6%) had household incomes of AUD$70 000 per annum or higher, whereas 5573 (11.9%) had an income of AUD$19 999 per annum or lower. University degree(s) were held by 16 398 (35.1%), 26 040 (55.7%) were employed, 16 762 (35.8%) were retired, and 10 236 (21.9%) were not in a couple. A total of 9822 (21.0%) had 30% or more total green land cover within 1.6 km from home compared with 11 957 (25.6%) who had 30% or more tree canopy and 2038 (4.4%) who had 30% or more grass within the same distance from home. A total of 1580 (3.4%) had 10% or more of the area within 1.6 km covered in other forms of low-lying vegetation.

At baseline, 5.1% of 37 775 reported a high risk of psychological distress, 16.0% of 46 786 reported depression or anxiety, and 9.0% of 45 577 reported fair to poor self-rated health (Table 1, Table 2, and Table 3). In the nonaffirmative sample at baseline, an additional 3.3% of 32 991 experienced psychological distress incidence, 7.5% of 39 277 experienced depression or anxiety incidence, and 7.3% of 40 741 experienced fair to poor self-rated health incidence by follow-up. Differences in prevalent and incident outcomes were found between sexes. For example, women compared with men had higher incidence of psychological distress (636 [3.7%] vs 440 [2.8%]) and physician-diagnosed depression or anxiety (1793 [8.9%] vs 1139 [6.0%]), but men had higher incidence of fair to poor general health compared with women (1502 [8.0%] to 1465 [6.7%]). The patterning of most outcomes at baseline and follow-up were lower among people with more total green space and more tree canopy nearby. Deviations from this pattern were incidence of depression or anxiety and fair to poor general health in association with total green space provision. Prevalent and incident outcomes appeared to be more common where there was more grass within 1.6 km. No consistent patterning of the health outcomes was noticeable with respect to low-lying vegetation.

**Table 1.** Cross-tabulation of Kessler 10-Item Psychological Distress Scale Data Across Potential Markers of Confounding and Green Space Variables

Variable	10-Item Kessler Psychological Distress Scale					
	Prevalence			Incidence		
	Value	χ^2^ Value	*P* Value	Value	χ^2^ Value	*P* Value
Total No. (% affirmative responses)	37 775 (5.1)	NA	NA	32 991 (3.3)	NA	NA
Sex						
Male		38.58	<.001		18.27	<.001
Subtotal, No.	17 629			15 602		
Affirmative responses, % (95% CI)	4.38 (4.09-4.69)			2.82 (2.57-3.09)		
Female						
Subtotal, No.	20 146			17 389		
Affirmative responses, % (95% CI)	5.79 (5.48-6.12)			3.66 (3.39-3.95)		
Age group, y						
45-54		215.87	<.001		41.98	<.001
Subtotal, No.	12 821			11 411		
Affirmative responses, % (95% CI)	7.17 (6.73-7.63)			4.07 (3.72-4.44)		
55-64						
Subtotal, No.	13 686			12 252		
Affirmative responses, % (95% CI)	4.99 (4.64-5.37)			2.74 (2.47-3.05)		
65-74						
Subtotal, No.	7228			6211		
Affirmative responses, % (95% CI)	2.95 (2.58-3.36)			2.66 (2.28-3.09)		
≥75						
Subtotal, No.	4040			3117		
Affirmative responses, % (95% CI)	3.07 (2.58-3.65)			3.56 (2.96-4.27)		
Annual household income, AUD$^a^						
0-19 999		319.40	<.001		84.69	<.001
Subtotal, No.	4089			3082		
Affirmative responses, % (95% CI)	10.49 (9.59-11.47)			5.22 (4.49-6.07)		
20 000-29 999						
Subtotal, No.	2510			2084		
Affirmative responses, % (95% CI)	6.14 (5.26-7.14)			4.32 (3.53-5.28)		
30 000-39 999						
Subtotal, No.	2469			2106		
Affirmative responses, % (95% CI)	5.83 (4.97-6.83)			3.32 (2.64-4.18)		
40 000-49 999						
Subtotal, No.	2572			2263		
Affirmative responses, % (95% CI)	4.67 (3.91-5.55)			3.89 (3.17-4.77)		
50 000-69 999						
Subtotal, No.	4346			3932		
Affirmative responses, % (95% CI)	4.49 (3.91-5.14)			3.05 (2.56-3.64)		
≥70 000						
Subtotal, No.	15 072			13 891		
Affirmative responses, % (95% CI)	3.68 (3.39-3.99)			2.41 (2.17-2.68)		
Not stated						
Subtotal, No.	6717			5633		
Affirmative responses, % (95% CI)	5.11 (4.60-5.66)			3.76 (3.30-4.29)		
Highest educational qualification						
None		166.02	<.001		86.77	<.001
Subtotal, No.	2103			1597		
Affirmative responses, % (95% CI)	9.99 (8.78-11.34)			6.39 (5.29-7.70)		
School						
Subtotal, No.	6218			5208		
Affirmative responses, % (95% CI)	5.92 (5.36-6.53)			3.88 (3.39-4.44)		
High school						
Subtotal, No.	3529			3067		
Affirmative responses, % (95% CI)	5.72 (5.00-6.54)			3.23 (2.66-3.92)		
Trade						
Subtotal, No.	3274			2762		
Affirmative responses, % (95% CI)	5.31 (4.60-6.14)			3.69 (3.05-4.46)		
Certificate or diploma						
Subtotal, No.	8479			7519		
Affirmative responses, % (95% CI)	4.86 (4.42-5.34)			3.39 (3.01-3.83)		
University						
Subtotal, No.	13 843			12 586		
Affirmative responses, % (95% CI)	3.92 (3.61-4.26)			2.42 (2.17-2.71)		
Not stated						
Subtotal, No.	329			252		
Affirmative responses, % (95% CI)	9.12 (6.45-12.75)			4.37 (2.43-7.72)		
Economic status						
Employed		1300.00	<.001		104.59	<.001
Subtotal, No.	21 686			19 669		
Affirmative responses, % (95% CI)	4.66 (4.38-4.95)			3.13 (2.90-3.38)		
Retired						
Subtotal, No.	13 091			11 027		
Affirmative responses, % (95% CI)	3.72 (3.41-4.06)			2.97 (2.67-3.31)		
Unemployed						
Subtotal, No.	507			367		
Affirmative responses, % (95% CI)	19.13 (15.94-22.79)			8.72 (6.23-12.08)		
Unpaid work						
Subtotal, No.	509			429		
Affirmative responses, % (95% CI)	7.27 (5.31-9.88)			3.03 (1.77-5.15)		
Disabled						
Subtotal, No.	431			237		
Affirmative responses, % (95% CI)	38.75 (34.25-43.44)			11.81 (8.28-16.59)		
Homemaker						
Subtotal, No.	1171			975		
Affirmative responses, % (95% CI)	8.45 (6.99-10.19)			4.21 (3.11-5.66)		
Other (eg, study)						
Subtotal, No.	380			287		
Affirmative responses, % (95% CI)	11.05 (8.27-14.63)			6.27 (3.98-9.74)		
Couple status						
Not in a couple		205.97	<.001		21.43	<.001
Subtotal, No.	7998			6572		
Affirmative responses, % (95% CI)	8.28 (7.69-8.90)			4.17 (3.71-4.68)		
In a couple						
Subtotal, No.	29 777			26 419		
Affirmative responses, % (95% CI)	4.29 (4.06-4.52)			3.04 (2.84-3.25)		
Total green space, %						
0-4		36.07	<.001		23.43	<.001
Subtotal, No.	586			491		
Affirmative responses, % (95% CI)	7.68 (5.78-10.13)			6.52 (4.64-9.07)		
5-9						
Subtotal, No.	8906			7715		
Affirmative responses, % (95% CI)	5.66 (5.20-6.16)			3.41 (3.03-3.84)		
10-19						
Subtotal, No.	9983			8672		
Affirmative responses, % (95% CI)	5.34 (4.91-5.80)			3.42 (3.06-3.83)		
20-29						
Subtotal, No.	10 296			8958		
Affirmative responses, % (95% CI)	5.24 (4.82-5.68)			3.19 (2.85-3.58)		
≥30						
Subtotal, No.	8004			7155		
Affirmative responses, % (95% CI)	3.97 (3.57-4.42)			2.77 (2.41-3.17)		
Tree canopy, %						
0-9		184.07	<.001		53.32	<.001
Subtotal, No.	3933			3283		
Affirmative responses, % (95% CI)	8.39 (7.56-9.30)			4.60 (3.93-5.37)		
10-19						
Subtotal, No.	14 403			12 400		
Affirmative responses, % (95% CI)	5.97 (5.60-6.37)			3.78 (3.46-4.13)		
20-29						
Subtotal, No.	9610			8450		
Affirmative responses, % (95% CI)	4.41 (4.02-4.84)			2.85 (2.52-3.23)		
≥30						
Subtotal, No.	9829			8858		
Affirmative responses, % (95% CI)	3.31 (2.97-3.68)			2.43 (2.13-2.77)		
Grass, %						
0-4		103.69	<.001		28.62	<.001
Subtotal, No.	4671			4177		
Affirmative responses, % (95% CI)	3.75 (3.24-4.33)			2.99 (2.52-3.55)		
5-9						
Subtotal, No.	15 792			13 950		
Affirmative responses, % (95% CI)	4.26 (3.95-4.58)			2.77 (2.51-3.06)		
10-19						
Subtotal, No.	9647			8352		
Affirmative responses, % (95% CI)	5.97 (5.52-6.46)			3.60 (3.22-4.03)		
20-29						
Subtotal, No.	6039			5141		
Affirmative responses, % (95% CI)	6.41 (5.82-7.05)			4.18 (3.67-4.76)		
≥30						
Subtotal, No.	1626			1371		
Affirmative responses, % (95% CI)	7.93 (6.72-9.35)			3.50 (2.65-4.62)		
Low-lying vegetation, %						
0-4		0.44	.81		3.65	.16
Subtotal, No.	24 188			21 127		
Affirmative responses, % (95% CI)	5.08 (4.81-5.36)			3.12 (2.90-3.37)		
5-9						
Subtotal, No.	12 326			10 753		
Affirmative responses, % (95% CI)	5.23 (4.85-5.64)			3.49 (3.16-3.85)		
≥10						
Subtotal, No.	1261			1111		
Affirmative responses, % (95% CI)	5.23 (4.13-6.61)			3.69 (2.73-4.97)		

Abbreviation: NA, not applicable.

^a^ To convert AUD to USD, divide by 1.44.

**Table 2.** Self-reported Physician-Diagnosed Depression or Anxiety Data Across Potential Markers of Confounding and Green Space Variables

Variable	Self-reported Physician-Diagnosed Depression or Anxiety					
	Prevalence			Incidence		
	Value	χ^2^ Value	*P* Value	Value	χ^2^ Value	*P* Value
Total No. (% affirmative responses)	46 786 (16.0)	NA	NA	39 277 (7.5)	NA	NA
Sex		626.40	<.001		124.52	<.001
Male						
Subtotal, No.	21 633			19 150		
Affirmative responses, % (95% CI)	11.45 (11.04-11.89)			5.95 (5.62-6.29)		
Female						
Subtotal, No.	25 153			20 127		
Affirmative responses, % (95% CI)	19.97 (19.48-20.47)			8.91 (8.52-9.31)		
Age group, y		292.22	<.001		99.60	<.001
45-54						
Subtotal, No.	15 443			12 526		
Affirmative responses, % (95% CI)	18.88 (18.27-19.51)			9.31 (8.81-9.83)		
55-64						
Subtotal, No.	16 604			13 807		
Affirmative responses, % (95% CI)	16.83 (16.27-17.41)			7.05 (6.64-7.49)		
65-74						
Subtotal, No.	9178			7952		
Affirmative responses, % (95% CI)	13.34 (12.66-14.05)			5.97 (5.47-6.52)		
≥75						
Subtotal, No.	5561			4992		
Affirmative responses, % (95% CI)	10.18 (9.41-11.00)			6.35 (5.71-7.06)		
Annual household income, AUD$^a^		115.29	<.001		50.29	<.001
0-19 999						
Subtotal, No.	5573			4438		
Affirmative responses, % (95% CI)	20.31 (19.28-21.39)			8.09 (7.32-8.93)		
20 000-29 999						
Subtotal, No.	3261			2703		
Affirmative responses, % (95% CI)	17.11 (15.86-18.44)			7.40 (6.47-8.45)		
30 000-39 999						
Subtotal, No.	3114			2576		
Affirmative responses, % (95% CI)	17.21 (15.93-18.58)			7.26 (6.32-8.33)		
40 000-49 999						
Subtotal, No.	3190			2678		
Affirmative responses, % (95% CI)	16.05 (14.82-17.37)			6.68 (5.80-7.69)		
50 000-69 999						
Subtotal, No.	5347			4468		
Affirmative responses, % (95% CI)	16.44 (15.47-17.46)			7.92 (7.17-8.75)		
≥70 000						
Subtotal, No.	17 611			14 975		
Affirmative responses, % (95% CI)	14.96 (14.44-15.50)			6.56 (6.17-6.97)		
Not stated						
Subtotal, No.	8690			7439		
Affirmative responses, % (95% CI)	14.37 (13.65-15.13)			9.02 (8.39-9.69)		
Highest educational qualification		69.92	<.001		54.75	<.001
None						
Subtotal, No.	2927			2372		
Affirmative responses, % (95% CI)	18.89 (17.52-20.35)			10.46 (9.29-11.75)		
School						
Subtotal, No.	8054			6731		
Affirmative responses, % (95% CI)	16.41 (15.62-17.24)			8.28 (7.64-8.96)		
High school						
Subtotal, No.	4419			3729		
Affirmative responses, % (95% CI)	15.59 (14.55-16.69)			7.05 (6.27-7.92)		
Trade						
Subtotal, No.	4170			3654		
Affirmative responses, % (95% CI)	12.33 (11.36-13.36)			7.01 (6.22-7.88)		
Certificate or diploma						
Subtotal, No.	10 366			8605		
Affirmative responses, % (95% CI)	16.98 (16.27-17.71)			7.69 (7.15-8.28)		
University						
Subtotal, No.	16 398			13 811		
Affirmative responses, % (95% CI)	15.77 (15.22-16.34)			6.62 (6.22-7.04)		
Not stated						
Subtotal, No.	452			375		
Affirmative responses, % (95% CI)	17.04 (13.84-20.79)			8.53 (6.10-11.82)		
Economic status		575.25	<.001		78.15	<.001
Employed						
Subtotal, No.	26 040			21 936		
Affirmative responses, % (95% CI)	15.75 (15.32-16.20)			7.47 (7.13-7.82)		
Retired						
Subtotal, No.	16 762			14 350		
Affirmative responses, % (95% CI)	14.36 (13.84-14.90)			6.80 (6.40-7.23)		
Unemployed						
Subtotal, No.	645			470		
Affirmative responses, % (95% CI)	27.13 (23.84-30.70)			11.70 (9.09-14.94)		
Unpaid work						
Subtotal, No.	663			536		
Affirmative responses, % (95% CI)	19.16 (16.33-22.33)			10.07 (7.80-12.93)		
Disabled						
Subtotal, No.	566			288		
Affirmative responses, % (95% CI)	48.94 (44.83-53.06)			17.36 (13.40-22.19)		
Homemaker						
Subtotal, No.	1541			1242		
Affirmative responses, % (95% CI)	19.40 (17.50-21.45)			8.86 (7.40-10.57)		
Other (eg, study)						
Subtotal, No.	569			455		
Affirmative responses, % (95% CI)	20.04 (16.94-23.53)			10.77 (8.23-13.97)		
Couple status		383.95	<.001		26.98	<.001
Not in a couple						
Subtotal, No.	10 236			7948		
Affirmative responses, % (95% CI)	22.31 (21.52-23.13)			8.83 (8.23-9.48)		
In a couple						
Subtotal, No.	36 550			31 329		
Affirmative responses, % (95% CI)	14.27 (13.92-14.64)			7.12 (6.84-7.41)		
Total green space, %		12.80	.012		13.47	.009
0-4						
Subtotal, No.	741			604		
Affirmative responses, % (95% CI)	18.49 (15.85-21.45)			6.29 (4.61-8.53)		
5-9						
Subtotal, No.	11 056			9203		
Affirmative responses, % (95% CI)	16.75 (16.07-17.46)			7.87 (7.33-8.43)		
10-19						
Subtotal, No.	12 455			10 508		
Affirmative responses, % (95% CI)	15.59 (14.97-16.24)			7.27 (6.79-7.78)		
20-29						
Subtotal, No.	12 712			10 651		
Affirmative responses, % (95% CI)	16.21 (15.57-16.86)			7.93 (7.44-8.46)		
≥30						
Subtotal, No.	9822			8311		
Affirmative responses, % (95% CI)	15.37 (14.67-16.10)			6.75 (6.23-7.31)		
Tree canopy, %		49.24	<.001		39.25	<.001
0-9						
Subtotal, No.	4972			4104		
Affirmative responses, % (95% CI)	17.42 (16.39-18.50)			8.77 (7.94-9.68)		
10-19						
Subtotal, No.	18 041			14 951		
Affirmative responses, % (95% CI)	17.11 (16.57-17.67)			8.17 (7.74-8.62)		
20-29						
Subtotal, No.	11 816			9988		
Affirmative responses, % (95% CI)	15.45 (14.81-16.12)			6.70 (6.22-7.21)		
≥30						
Subtotal, No.	11 957			10 234		
Affirmative responses, % (95% CI)	14.40 (13.78-15.04)			6.65 (6.19-7.15)		
Grass, %		39.30	<.001		29.19	<.001
0-4						
Subtotal, No.	5706			4787		
Affirmative responses, % (95% CI)	16.09 (15.16-17.06)			7.06 (6.37-7.82)		
5-9						
Subtotal, No.	19 359			16 433		
Affirmative responses, % (95% CI)	15.10 (14.60-15.61)			6.80 (6.42-7.19)		
10-19						
Subtotal, No.	12 072			10 134		
Affirmative responses, % (95% CI)	16.04 (15.39-16.70)			7.85 (7.35-8.39)		
20-29						
Subtotal, No.	7611			6273		
Affirmative responses, % (95% CI)	17.55 (16.72-18.42)			8.48 (7.82-9.20)		
≥30						
Subtotal, No.	2038			1650		
Affirmative responses, % (95% CI)	19.04 (17.39-20.80)			9.03 (7.74-10.51)		
Low-lying vegetation, %		2.05	.36		1.86	.40
0-4						
Subtotal, No.	29 840			25 097		
Affirmative responses, % (95% CI)	15.88 (15.47-16.30)			7.33 (7.02-7.66)		
5-9						
Subtotal, No.	15 366			12 847		
Affirmative responses, % (95% CI)	16.37 (15.80-16.97)			7.68 (7.23-8.16)		
≥10						
Subtotal, No.	1580			1333		
Affirmative responses, % (95% CI)	15.63 (13.92-17.51)			7.88 (6.55-9.45)		

Abbreviation: NA, not applicable.

^a^ To convert AUD to USD, divide by 1.44.

**Table 3.** Cross-tabulation of Self-rated Fair or Poor General Health Across Potential Markers of Confounding and Green Space Variables

Variable	Self-rated Fair or Poor General Health					
	Prevalence			Incidence		
	Value	χ^2^ Value	*P* Value	Value	χ^2^ Value	*P* Value
Total No. (% affirmative responses)	45 577 (9.0)	NA	NA	40 741 (7.3)	NA	NA
Sex						
Male		4.55	.03		26.77	<.001
Subtotal, No.	21 166			18 767		
Affirmative responses, % (95% CI)	9.26 (8.88-9.66)			8.00 (7.62-8.40)		
Female						
Subtotal, No.	24 411			21 974		
Affirmative responses, % (95% CI)	8.69 (8.35-9.05)			6.67 (6.34-7.00)		
Age group, y						
45-54		165.35	<.001		971.70	<.001
Subtotal, No.	15 119			13 821		
Affirmative responses, % (95% CI)	7.83 (7.41-8.27)			4.82 (4.47-5.19)		
55-64						
Subtotal, No.	16 200			14 671		
Affirmative responses, % (95% CI)	8.22 (7.80-8.65)			5.45 (5.09-5.83)		
65-74						
Subtotal, No.	8928			7864		
Affirmative responses, % (95% CI)	9.59 (8.99-10.22)			9.10 (8.49-9.76)		
≥75						
Subtotal, No.	5330			4385		
Affirmative responses, % (95% CI)	13.36 (12.47-14.30)			17.92 (16.82-19.09)		
Annual household income, AUD$^a^						
0-19 999		1300.00	<.001		761.19	<.001
Subtotal, No.	5428			4170		
Affirmative responses, % (95% CI)	20.49 (19.43-21.58)			15.25 (14.19-16.38)		
20 000-29 999						
Subtotal, No.	3205			2741		
Affirmative responses, % (95% CI)	12.23 (11.14-13.41)			11.46 (10.32-12.70)		
30 000-39 999						
Subtotal, No.	3067			2754		
Affirmative responses, % (95% CI)	8.41 (7.48-9.45)			8.75 (7.75-9.87)		
40 000-49 999						
Subtotal, No.	3152			2844		
Affirmative responses, % (95% CI)	8.22 (7.31-9.23)			7.81 (6.88-8.85)		
50 000-69 999						
Subtotal, No.	5298			4856		
Affirmative responses, % (95% CI)	6.96 (6.31-7.68)			5.87 (5.24-6.57)		
≥70 000						
Subtotal, No.	17 504			16 463		
Affirmative responses, % (95% CI)	4.90 (4.59-5.23)			4.06 (3.77-4.37)		
Not stated						
Subtotal, No.	7923			6913		
Affirmative responses, % (95% CI)	10.55 (9.89-11.25)			8.69 (8.05-9.38)		
Highest educational qualification						
None		752.66	<.001		419.92	<.001
Subtotal, No.	2785			2178		
Affirmative responses, % (95% CI)	19.35 (17.93-20.86)			14.33 (12.92-15.86)		
School						
Subtotal, No.	7794			6762		
Affirmative responses, % (95% CI)	11.42 (10.73-12.14)			8.99 (8.33-9.70)		
High school						
Subtotal, No.	4297			3756		
Affirmative responses, % (95% CI)	10.80 (9.90-11.76)			8.23 (7.39-9.15)		
Trade						
Subtotal, No.	4071			3511		
Affirmative responses, % (95% CI)	11.74 (10.79-12.77)			10.60 (9.62-11.66)		
Certificate or diploma						
Subtotal, No.	10 153			9240		
Affirmative responses, % (95% CI)	7.51 (7.01-8.03)			6.62 (6.13-7.15)		
University						
Subtotal, No.	16 061			14 951		
Affirmative responses, % (95% CI)	5.55 (5.20-5.91)			4.74 (4.41-5.09)		
Not stated						
Subtotal, No.	416			343		
Affirmative responses, % (95% CI)	14.18 (11.15-17.88)			13.41 (10.19-17.45)		
Economic status						
Employed		2300.00	<.001		584.94	<.001
Subtotal, No.	25 482			23 679		
Affirmative responses, % (95% CI)	5.96 (5.67-6.25)			4.81 (4.54-5.09)		
Retired						
Subtotal, No.	16 263			14 055		
Affirmative responses, % (95% CI)	11.06 (10.58-11.55)			10.94 (10.44-11.47)		
Unemployed						
Subtotal, No.	616			470		
Affirmative responses, % (95% CI)	22.24 (19.13-25.70)			12.13 (9.47-15.41)		
Unpaid work						
Subtotal, No.	646			586		
Affirmative responses, % (95% CI)	7.89 (6.05-10.24)			6.48 (4.75-8.79)		
Disabled						
Subtotal, No.	539			209		
Affirmative responses, % (95% CI)	60.11 (55.91-64.17)			20.57 (15.62-26.61)		
Homemaker						
Subtotal, No.	1498			1307		
Affirmative responses, % (95% CI)	11.35 (9.84-13.06)			7.35 (6.05-8.89)		
Other (eg, study)						
Subtotal, No.	533			435		
Affirmative responses, % (95% CI)	15.95 (13.08-19.31)			12.87 (10.04-16.37)		
Couple status						
Not in a couple		83.98	<.001		35.48	<.001
Subtotal, No.	9876			8395		
Affirmative responses, % (95% CI)	13.05 (12.40-13.73)			10.29 (9.66-10.96)		
In a couple						
Subtotal, No.	35 701			32 346		
Affirmative responses, % (95% CI)	7.83 (7.55-8.11)			6.50 (6.24-6.78)		
χ^2^ Value	259.02			141.81		
*P* value	<.001			<.001		
Total green space, %						
0-4						
Subtotal, No.	722			611		
Affirmative responses, % (95% CI)	13.02 (10.75-15.68)			5.40 (3.86-7.50)		
5-9						
Subtotal, No.	10 734			9507		
Affirmative responses, % (95% CI)	9.74 (9.19-10.31)			7.49 (6.98-8.04)		
10-19						
Subtotal, No.	12 102			10 716		
Affirmative responses, % (95% CI)	9.49 (8.98-10.03)			8.03 (7.53-8.56)		
20-29						
Subtotal, No.	12 423			11 086		
Affirmative responses, % (95% CI)	9.22 (8.72-9.74)			7.52 (7.05-8.03)		
≥30						
Subtotal, No.	9596			8821		
Affirmative responses, % (95% CI)	6.77 (6.29-7.29)			5.99 (5.51-6.50)		
Tree canopy, %						
0-9		437.08	<.001		123.85	<.001
Subtotal, No.	4821			4056		
Affirmative responses, % (95% CI)	14.23 (13.27-15.24)			10.36 (9.45-11.33)		
10-19						
Subtotal, No.	17 513			15 309		
Affirmative responses, % (95% CI)	10.83 (10.37-11.30)			8.13 (7.71-8.58)		
20-29						
Subtotal, No.	11 525			10 478		
Affirmative responses, % (95% CI)	7.30 (6.84-7.79)			6.53 (6.07-7.02)		
≥30						
Subtotal, No.	11 718			10 898		
Affirmative responses, % (95% CI)	5.63 (5.23-6.06)			5.67 (5.25-6.12)		
Grass, %						
0-4		247.58	<.001		87.45	
Subtotal, No.	5561			5139		
Affirmative responses, % (95% CI)	6.28 (5.67-6.94)			5.66 (5.06-6.33)		
5-9						
Subtotal, No.	18 865			17 143		
Affirmative responses, % (95% CI)	7.42 (7.05-7.80)			6.43 (6.07-6.81)		
10-19						
Subtotal, No.	11 778			10 385		
Affirmative responses, % (95% CI)	10.07 (9.54-10.63)			8.10 (7.59-8.64)		
20-29						
Subtotal, No.	7389			6370		
Affirmative responses, % (95% CI)	12.15 (11.43-12.92)			9.14 (8.45-9.87)		
≥30						
Subtotal, No.	1984			1704		
Affirmative responses, % (95% CI)	12.65 (11.26-14.19)			8.86 (7.60-10.31)		<.001
Low-lying vegetation, %						
0-4		5.10	.81		8.62	.01
Subtotal, No.	29 080			26 038		
Affirmative responses, % (95% CI)	8.74 (8.42-9.07)			7.08 (6.77-7.40)		
5-9						
Subtotal, No.	14 958			13 334		
Affirmative responses, % (95% CI)	9.29 (8.84-9.77)			7.78 (7.34-8.25)		
≥10						
Subtotal, No.	1539			1369		
Affirmative responses, % (95% CI)	9.81 (8.42-11.40)			6.28 (5.11-7.70)		

Abbreviation: NA, not applicable.

^a^ To convert AUD to USD, divide by 1.44.

The patterns described above generally held after adjustment for confounding in multilevel models (Figure and eTables 4-9 in the Supplement). Consistently lower odds of prevalent and incident psychological distress and fair to poor general health was associated with exposure to more tree canopy nearby. For example, among those with 30% or more tree canopy compared with 0% to 9%, the odds of incident psychological distress were 0.69 (95% CI, 0.54-0.88) and of incident fair to poor general health were 0.67 (95% CI, 0.57-0.80). The odds of incident depression or anxiety were also lower with more tree canopy, but these were not statistically significant (odds ratio, 0.86; 95% CI, 0.74 to >1.00). Inconsistent results across the outcomes with respect to total green space may have been associated with the availability of grass without tree canopy, for which the odds of prevalent and incident outcomes but for incident psychological distress were higher. For example, the odds of incident fair to poor general health were 1.47 (95% CI, 1.12-1.91) and of prevalent psychological distress were 1.71 (95% CI, 1.25-2.28) for people with 30% or more grass nearby compared with those with 0% to 4%. As with the descriptive analyses, no consistent associations were found for low-lying vegetation.

**Figure.**
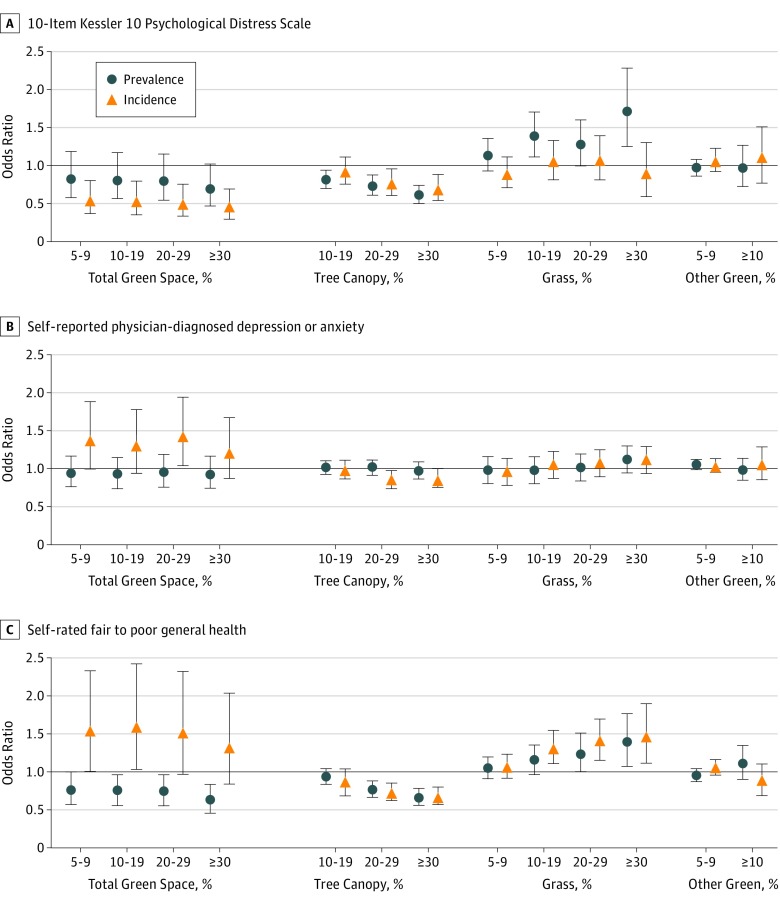
Associations Between Types of Green Space and Psychological Distress, Depression or Anxiety, and Fair to Poor General Health Findings are adjusted for confounding in multilevel logistic regressions. For total green space, grass, and other green, the reference category is 0% to 4%; for tree canopy, the reference category is 0% to 9%. Error bars indicate 95% CIs.

## Discussion

The results of this longitudinal study may help enhance knowledge of the mental health benefits of green space within the context of a literature dominated by cross-sectional data and singular exposure measures.^25,32^ In line with some of the previous longitudinal research,^33^ total green space appeared to be associated with lower odds of incident psychological distress. In this study, exposure to tree canopy was associated with less prevalent and incident psychological distress and better self-rated general health; thus, provision of more tree canopy may be an effective option for supporting community mental health in urban greening strategies. Findings were otherwise limited or inconsistent for the physician-diagnosed measure of depression or anxiety. Health economic evaluation will be an important next step to discern to what extent tree canopy may be considered the most cost-effective means of urban greening for better mental health.

This study benefited from a large, residentially stable sample followed up for a mean of 6.2 years linked to objectively measured green space exposures. The focus on place of residence at baseline meant that the longitudinal analysis of incident cases tested lagged exposure of green space on each outcome, which helped to guard against bias induced by selective migration. However, we did not know where people lived before the baseline survey. Thus, we cannot rule out the contribution of selective migration to the cross-sectional analyses of baseline data focusing on prevalence; people already in better mental health may have moved to areas with more of the types of green space that they prefer, thus emphasizing the importance of the longitudinal analyses also conducted.

In terms of recommendations for decision-makers and policy influencers, the association found between the risk of psychological distress and fair to poor self-rated general health and a higher availability of tree canopy within 1.6 km is noteworthy. Population growth and the demand for more housing, amenities, and infrastructure in Sydney, Newcastle, and Wollongong is a challenge experienced in many other cities worldwide. Street trees in prime building locations are at a particular risk of being cut down. Shorn of tree canopy, sidewalk temperatures can be higher,^34^ sidewalks can seem noisier,^17^ and walkers along them are exposed to more air pollution.^35^ Street trees provide a valuable aesthetic use, such as providing pleasant views from the side of an adjacent street.

Biodiversity (eg, birds) may also play an interrelated role. A recent meta-analysis^36^ reported that tree canopy is more supportive of biodiversity than open grasslands. Furthermore, a previous study^16^ suggested that higher levels of biodiversity, rather than the amount of green space, was associated with more favorable levels of psychological well-being. A similar finding was also reported recently in the United Kingdom.^37^ For people engaging in passive recreation relating to biodiversity, such as bird-watching, or other forms of recreation, such as walking, tree canopy is likely to be an important part of that experience and the benefits that accrue for well-being.^7,8,9,10,11,12,38,39^

The evidence also suggests that more land use dedicated to grass without tree canopy may not support mental health. This finding ought not be interpreted as evidence for removing existing grassy areas or defunding the planting of new open grassy areas because the result in this study may be confounded with other factors that are detrimental to mental health. Results from a previous study^40^ that observed higher mortality rates in US cities that contain more green space may hold some clues. This result may be associated with urban sprawl and related factors, such as longer distances and a lack of public infrastructure necessitating reliance on car travel, with grass accounting for a large amount of the land use in between.^40^ The same issue may also apply to the current study. Another potential contributor to this result is evidence suggesting that humans prefer to visit green spaces with more complex vegetation,^19^ whereas plain grassy areas may be not be particularly attractive to walking among older adults.^21^ The benefits of heat islands may also be less mitigated by grassy land use.^24^

A related issue is that although some types of green space may be set apart geographically, combinations thereof are likely to be more common (eg, an open grassy area with low-lying vegetation and tree canopy along the perimeter). The potential for combinations of different types of green space may be addressed in part by the total green space measure and also help to explain some of the larger ORs obtained for psychological distress prevalence compared with those for tree canopy only. Isotemporal substitution models and discrete choice experiments may offer potential avenues for future research to reveal how much of which type of green space best supports mental health within the presence of other green space types. This contextual dependency may also be expanded to other spatial physical and social phenomena. For example, particular types and combinations of green space may also help or harm feelings of community safety and perceptions of (or actual) crime rates, with well-known implications for mental health.^26,41^

### Limitations

These results should be interpreted within the context of the limitations, including the use of self-reported health outcomes. It is plausible that results were inconsistent for physician-diagnosed depression or anxiety because its reporting may be affected by the stigma associated with mental ill-health.^42,43^ Future research that involves prescription data and biomarkers (eg, cortisol) would be valuable. The green space data were the best available but measured in 2016 and not beforehand, which is a limitation because green space availability may have decreased in some areas over time. This limitation may mean that our results are underestimates of the true associations. Furthermore, no information was available on duration of residence before baseline, which may be a potential effect modifier.

## Conclusions

Our findings suggest that urban greening strategies with a remit for supporting community mental health should prioritize the protection and restoration of urban tree canopy. In addition, the promotion of equal access to tree canopy may provide greater equity in mental health.

## Notes

Supplement. **eFigure.** Flowchart for the Sample **eTable 1.** Cross-tabulation of Missing Kessler 10 Psychological Distress Scale Data at Baseline and Follow-up **eTable 2.** Cross-tabulation of Missing Self-reported Physician-Diagnosed Depression or Anxiety Data at Baseline and Follow-up **eTable 3.** Cross-tabulation of Missing Self-rated Fair or Poor General Health Data at Baseline and Follow-up **eTable 4.** Associations Between Total Green Space, Green Space Type, and Prevalent Kessler 10 Psychological Distress Adjusted for Confounding in Multilevel Logistic Regressions Estimated With Markov Chain Monte Carlo (MCMC) **eTable 5.** Associations Between Total Green Space, Green Space Type, and Incident Kessler 10 Psychological Distress Adjusted for Confounding in Multilevel Logistic Regressions Estimated With Markov Chain Monte Carlo (MCMC) **eTable 6.** Associations Between Total Green Space, Green Space Type, and Prevalent Self-reported Physician-Diagnosed Depression or Anxiety Adjusted for Confounding in Multilevel Logistic Regressions Estimated With Markov Chain Monte Carlo (MCMC) **eTable 7.** Associations Between Total Green Space, Green Space Type, and Incident Self-reported Physician-Diagnosed Depression or Anxiety Adjusted for Confounding in Multilevel Logistic Regressions Estimated With Markov Chain Monte Carlo (MCMC) **eTable 8.** Associations Between Total Green Space, Green Space Type, and Prevalent Missing Self-rated Fair or Poor General Health Adjusted for Confounding in Multilevel Logistic Regressions Estimated With Markov Chain Monte Carlo (MCMC) **eTable 9.** Associations Between Total Green Space, Green Space Type, and Incident Missing Self-rated Fair or Poor General Health Adjusted for Confounding in Multilevel Logistic Regressions Estimated With Markov Chain Monte Carlo (MCMC)
