# Supplementary material for: Association of Urban Green Space With Mental Health and General Health Among Adults in Australia
Source: JAMA Netw Open. 2019 Jul 26;2(7):e198209. doi: 10.1001/jamanetworkopen.2019.8209 (PMC6661720; doi:10.1001/jamanetworkopen.2019.8209)
Supplement: Supplement. — eFigure. Flowchart for the Sample eTable 1. Cross-tabulation of Missing Kessler 10 Psychological Distress Scale Data at Baseline and Follow-up eTable 2. Cross-tabulation of Missing Self-reported Physician-Diagnosed Depression or Anxiety Data at Baseline and Follow-up eTable 3. Cross-tabulation of Missing Self-rated Fair or Poor General Health Data at Baseline and Follow-up eTable 4. Associations Between Total Green Space, Green Space Type, and Prevalent Kessler 10 Psychological Distress Adjusted for Confounding in Multilevel Logistic Regressions Estimated With Markov Chain Monte Carlo (MCMC) eTable 5. Associations Between Total Green Space, Green Space Type, and Incident Kessler 10 Psychological Distress Adjusted for Confounding in Multilevel Logistic Regressions Estimated With Markov Chain Monte Carlo (MCMC) eTable 6. Associations Between Total Green Space, Green Space Type, and Prevalent Self-reported Physician-Diagnosed Depression or Anxiety Adjusted for Confounding in Multilevel Logistic Regressions Estimated With Markov Chain Monte Carlo (MCMC) eTable 7. Associations Between Total Green Space, Green Space Type, and Incident Self-reported Physician-Diagnosed Depression or Anxiety Adjusted for Confounding in Multilevel Logistic Regressions Estimated With Markov Chain Monte Carlo (MCMC) eTable 8. Associations Between Total Green Space, Green Space Type, and Prevalent Missing Self-rated Fair or Poor General Health Adjusted for Confounding in Multilevel Logistic Regressions Estimated With Markov Chain Monte Carlo (MCMC) eTable 9. Associations Between Total Green Space, Green Space Type, and Incident Missing Self-rated Fair or Poor General Health Adjusted for Confounding in Multilevel Logistic Regressions Estimated With Markov Chain Monte Carlo (MCMC) [file jamanetwopen-2-e198209-s001.pdf]

Supplementary Online Content

Astell-Burt T, Feng X. Association of urban green space with mental health and general health among adults in Australia. *JAMA Netw Open*. 2019;2(7):e198209. doi:10.1001/jamanetworkopen.2019.8209

eFigure. Flowchart for the Sample

eTable 1. Cross-tabulation of Missing Kessler 10 Psychological Distress Scale Data at Baseline and Follow-up

eTable 2. Cross-tabulation of Missing Self-reported Physician-Diagnosed Depression or Anxiety Data at Baseline and Follow-up

eTable 3. Cross-tabulation of Missing Self-rated Fair or Poor General Health Data at Baseline and Follow-up

eTable 4. Associations Between Total Green Space, Green Space Type, and Prevalent Kessler 10 Psychological Distress Adjusted for Confounding in Multilevel Logistic Regressions Estimated With Markov Chain Monte Carlo (MCMC)

eTable 5. Associations Between Total Green Space, Green Space Type, and Incident Kessler 10 Psychological Distress Adjusted for Confounding in Multilevel Logistic Regressions Estimated With Markov Chain Monte Carlo (MCMC)

eTable 6. Associations Between Total Green Space, Green Space Type, and Prevalent Self-reported Physician-Diagnosed Depression or Anxiety Adjusted for Confounding in Multilevel Logistic Regressions Estimated With Markov Chain Monte Carlo (MCMC)

eTable 7. Associations Between Total Green Space, Green Space Type, and Incident Self-reported Physician-Diagnosed Depression or Anxiety Adjusted for Confounding in Multilevel Logistic Regressions Estimated With Markov Chain Monte Carlo (MCMC)

eTable 8. Associations Between Total Green Space, Green Space Type, and Prevalent Missing Self-rated Fair or Poor General Health Adjusted for Confounding in Multilevel Logistic Regressions Estimated With Markov Chain Monte Carlo (MCMC)

eTable 9. Associations Between Total Green Space, Green Space Type, and Incident Missing Self-rated Fair or Poor General Health Adjusted for Confounding in Multilevel Logistic Regressions Estimated With Markov Chain Monte Carlo (MCMC)

This supplementary material has been provided by the authors to give readers additional information about their work.

eFigure. Flowchart for the Sample

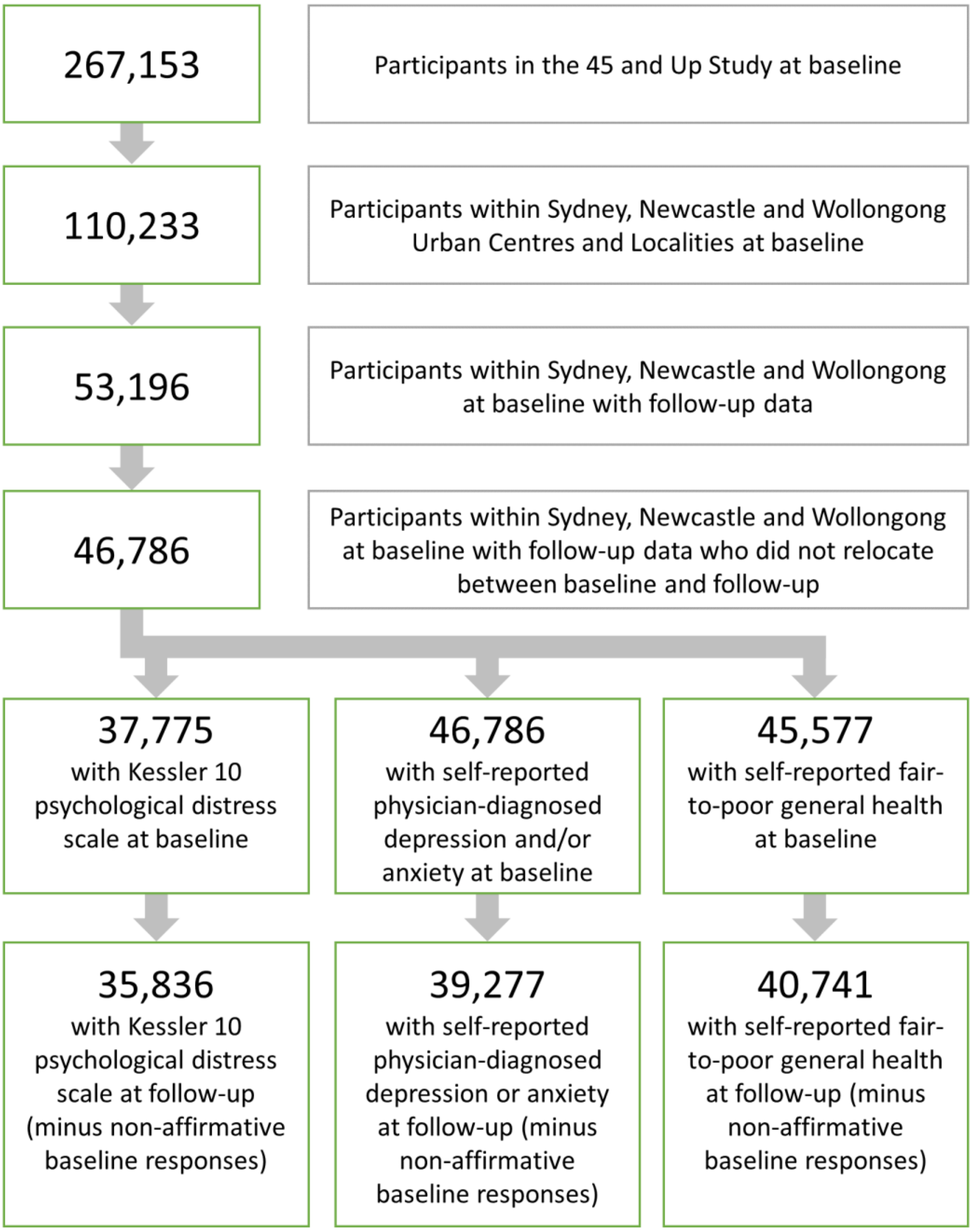

© 2019 Astell-Burt T et al. *JAMA Network Open.*

eTable 1: Cross-tabulation of missing Kessler 10 Psychological Distress Scale data at baseline and follow-up

	10-item Kessler Psychological Distress Scale	
	Prevalence	Incidence
Total (% missing responses)	46786 (19.26%)	35836 (7.94%)
	Subtotal (% affirmative responses, 95%CI)	
Gender		
Male	21,633 (18.51%, 18.00% to 19.03%)	16,857 (7.44%, 7.06% to 7.85%)
Female	25,153 (19.91%, 19.42% to 20.40%)	18,979 (8.38%, 7.99% to 8.78%)
Chi-square (p-value)	14.60 (p<0.001)	10.63 (p=0.001)
Age group		
45-54y	15,443 (16.98%, 16.39% to 17.58%)	11,902 (4.13%, 3.78% to 4.50%)
55-64y	16,604 (17.57%, 17.00% to 18.16%)	13,003 (5.78%, 5.39% to 6.19%)
65-74y	9,178 (21.25%, 20.42% to 22.10%)	7,015 (11.46%, 10.74% to 12.23%)
≥75y	5,561 (27.35%, 26.20% to 28.54%)	3,916 (20.40%, 19.17% to 21.69%)
Chi-square (p-value)	339.44 (p<0.001)	1,300.00 (p<0.001)
Annual household income		
$0-$19,999	5,573 (26.63%, 25.48% to 27.81%)	3,660 (15.79%, 14.65% to 17.01%)
$20,000-$29,999	3,261 (23.03%, 21.62% to 24.51%)	2,356 (11.54%, 10.32% to 12.90%)
$30,000-$39,999	3,114 (20.71%, 19.33% to 22.17%)	2,325 (9.42%, 8.30% to 10.68%)
$40,000-$49,999	3,190 (19.37%, 18.04% to 20.78%)	2,452 (7.71%, 6.72% to 8.83%)
$50,000-$69,999	5,347 (18.72%, 17.70% to 19.79%)	4,151 (5.28%, 4.64% to 6.00%)
$70,000+	17,611 (14.42%, 13.91% to 14.94%)	14,518 (4.32%, 4.00% to 4.66%)
Not stated	8,690 (22.70%, 21.84% to 23.60%)	6,374 (11.63%, 10.86% to 12.44%)
Chi-square (p-value)	561.53 (p<0.001)	777.05 (p<0.001)
Highest educational qualification		
None	2,927 (28.15%, 26.55% to 29.81%)	1,893 (15.64%, 14.07% to 17.34%)
School	8,054 (22.80%, 21.89% to 23.73%)	5,850 (10.97%, 10.20% to 11.80%)
High school	4,419 (20.14%, 18.98% to 21.35%)	3,327 (7.81%, 6.95% to 8.78%)
Trade	4,170 (21.49%, 20.27% to 22.76%)	3,100 (10.90%, 9.85% to 12.05%)
Certificate/Diploma	10,366 (18.20%, 17.47% to 18.96%)	8,067 (6.79%, 6.26% to 7.36%)
University	16,398 (15.58%, 15.03% to 16.14%)	13,300 (5.37%, 5.00% to 5.76%)
Not stated	452 (27.21%, 23.30% to 31.51%)	299 (15.72%, 12.01% to 20.31%)
Chi-square (p-value)	397.61 (p<0.001)	424.06 (p<0.001)
Economic status		
Employed	26,040 (16.72%, 16.27% to 17.18%)	20,676 (4.87%, 4.59% to 5.17%)
Retired	16,762 (21.90%, 21.28% to 22.53%)	12,604 (12.51%, 11.95% to 13.10%)
Unemployed	645 (21.40%, 18.40% to 24.73%)	410 (10.49%, 7.87% to 13.85%)
Unpaid work	663 (23.23%, 20.17% to 26.60%)	472 (9.11%, 6.82% to 12.06%)
Disabled	566 (23.85%, 20.52% to 27.54%)	264 (10.23%, 7.10% to 14.51%)
Homemaker	1,541 (24.01%, 21.94% to 26.21%)	1,072 (9.05%, 7.47% to 10.92%)
Other (e.g. study)	569 (33.22%, 29.46% to 37.20%)	338 (15.09%, 11.65% to 19.32%)

© 2019 Astell-Burt T et al. *JAMA Network Open.*

Chi-square (p-value)	293.07 (p<0.001)	658.88 (p<0.001)
Couple status		
Not in a couple	10,236 (21.86%, 21.07% to 22.68%)	7,336 (10.41%, 9.74% to 11.13%)
In a couple	36,550 (18.53%, 18.14% to 18.93%)	28,500 (7.30%, 7.01% to 7.61%)
Chi-square (p-value)	57.13 (p<0.001)	77.34 (p<0.001)
Total green space		
0-4%	741 (20.92%, 18.14% to 24.00%)	541 (9.24%, 7.07% to 11.99%)
5-9%	11,056 (19.45%, 18.72% to 20.19%)	8,402 (8.18%, 7.61% to 8.78%)
10-19%	12,455 (19.85%, 19.16% to 20.56%)	9,450 (8.23%, 7.70% to 8.80%)
20-29%	12,712 (19.01%, 18.33% to 19.70%)	9,757 (8.19%, 7.66% to 8.75%)
≥30%	9,822 (18.51%, 17.75% to 19.29%)	7,686 (6.91%, 6.36% to 7.50%)
Chi-square (p-value)	8.41 (p<0.001)	15.02 (p=0.005)
Tree canopy		
0-9%	4,972 (20.90%, 19.79% to 22.05%)	3,603 (8.88%, 8.00% to 9.86%)
10-19%	18,041 (20.17%, 19.59% to 20.76%)	13,543 (8.44%, 7.98% to 8.92%)
20-29%	11,816 (18.67%, 17.98% to 19.38%)	9,186 (8.01%, 7.47% to 8.59%)
≥30%	11,957 (17.80%, 17.12% to 18.49%)	9,504 (6.80%, 6.31% to 7.32%)
Chi-square (p-value)	37.18 (p<0.001)	26.05 (p<0.001)
Grass		
0-4%	5,706 (18.14%, 17.16% to 19.16%)	4,496 (7.10%, 6.38% to 7.88%)
5-9%	19,359 (18.43%, 17.89% to 18.98%)	15,120 (7.74%, 7.32% to 8.17%)
10-19%	12,072 (20.09%, 19.38% to 20.81%)	9,071 (7.93%, 7.39% to 8.50%)
20-29%	7,611 (20.65%, 19.76% to 21.58%)	5,652 (9.04%, 8.32% to 9.82%)
≥30%	2,038 (20.22%, 18.53% to 22.02%)	1,497 (8.42%, 7.11% to 9.93%)
Chi-square (p-value)	29.31 (p<0.001)	15.08 (p=0.005)
Low-lying vegetation		
0-4%	29,840 (18.94%, 18.50% to 19.39%)	22,960 (7.98%, 7.64% to 8.34%)
5-9%	15,366 (19.78%, 19.16% to 20.42%)	11,681 (7.94%, 7.47% to 8.45%)
≥10%	1,580 (20.19%, 18.28% to 22.24%)	1,195 (7.03%, 5.71% to 8.62%)
Chi-square (p-value)	5.54 (p=0.063)	1.42 (p=0.493)
95%CI: 95% Confidence Interval		

© 2019 Astell-Burt T et al. *JAMA Network Open.*

eTable 2: Cross-tabulation of missing self-reported physician-diagnosed depression or anxiety data at baseline and follow-up

	Self-reported physician-diagnosed depression or anxiety	
	Prevalence	Incidence
Total (% missing responses)	46786 (0.00%)	39277 (0.02%)
	Subtotal (% affirmative responses, 95%CI)	
Gender		
Male	21,633 (0.00%)	19,155 (0.03%, 0.01% to 0.06%)
Female	25,153 (0.00%)	20,130 (0.01%, 0.00% to 0.05%)
Chi-square (p-value)	.	0.60 (p=0.437)
Age group		
45-54y	15,443 (0.00%)	12,527 (0.01%, 0.00% to 0.06%)
55-64y	16,604 (0.00%)	13,809 (0.01%, 0.00% to 0.06%)
65-74y	9,178 (0.00%)	7,954 (0.03%, 0.01% to 0.10%)
≥75y	5,561 (0.00%)	4,995 (0.06%, 0.02% to 0.19%)
Chi-square (p-value)	.	5.13 (p=0.162)
Annual household income		
$0-$19,999	5,573 (0.00%)	4,441 (0.07%, 0.02% to 0.21%)
$20,000-$29,999	3,261 (0.00%)	2,703 (0.00%)
$30,000-$39,999	3,114 (0.00%)	2,578 (0.08%, 0.02% to 0.31%)
$40,000-$49,999	3,190 (0.00%)	2,678 (0.00%)
$50,000-$69,999	5,347 (0.00%)	4,468 (0.00%)
$70,000+	17,611 (0.00%)	14,976 (0.01%, 0.00% to 0.05%)
Not stated	8,690 (0.00%)	7,441 (0.03%, 0.01% to 0.11%)
Chi-square (p-value)	.	12.54 (p=0.051)
Highest educational qualification		
None	2,927 (0.00%)	2,374 (0.08%, 0.02% to 0.34%)
School	8,054 (0.00%)	6,732 (0.01%, 0.00% to 0.11%)
High school	4,419 (0.00%)	3,730 (0.03%, 0.00% to 0.19%)
Trade	4,170 (0.00%)	3,656 (0.05%, 0.01% to 0.22%)
Certificate/Diploma	10,366 (0.00%)	8,606 (0.01%, 0.00% to 0.08%)
University	16,398 (0.00%)	13,812 (0.01%, 0.00% to 0.05%)
Not stated	452 (0.00%)	375 (0.00%)
Chi-square (p-value)	.	8.62 (p=0.196)
Economic status		
Employed	26,040 (0.00%)	21,938 (0.01%, 0.00% to 0.04%)
Retired	16,762 (0.00%)	14,355 (0.03%, 0.01% to 0.08%)
Unemployed	645 (0.00%)	470 (0.00%)
Unpaid work	663 (0.00%)	536 (0.00%)
Disabled	566 (0.00%)	289 (0.35%, 0.05% to 2.42%)
Homemaker	1,541 (0.00%)	1,242 (0.00%)
Other (e.g. study)	569 (0.00%)	455 (0.00%)
Chi-square (p-value)	.	18.44 (p=0.005)

© 2019 Astell-Burt T et al. *JAMA Network Open.*

Couple status		
Not in a couple	10,236 (0.00%)	7,952 (0.05%, 0.02% to 0.13%)
In a couple	36,550 (0.00%)	31,333 (0.01%, 0.00% to 0.03%)
Chi-square (p-value)	.	4.39 (p=0.036)
Total green space		
0-4%	741 (0.00%)	604 (0.00%)
5-9%	11,056 (0.00%)	9,204 (0.01%, 0.00% to 0.08%)
10-19%	12,455 (0.00%)	10,513 (0.05%, 0.02% to 0.11%)
20-29%	12,712 (0.00%)	10,652 (0.01%, 0.00% to 0.07%)
≥30%	9,822 (0.00%)	8,312 (0.01%, 0.00% to 0.09%)
Chi-square (p-value)	.	5.26 (p=0.261)
Tree canopy		
0-9%	4,972 (0.00%)	4,106 (0.05%, 0.01% to 0.19%)
10-19%	18,041 (0.00%)	14,954 (0.02%, 0.01% to 0.06%)
20-29%	11,816 (0.00%)	9,990 (0.02%, 0.01% to 0.08%)
≥30%	11,957 (0.00%)	10,235 (0.01%, 0.00% to 0.07%)
Chi-square (p-value)	.	2.19 (p=0.535)
Grass		
0-4%	5,706 (0.00%)	4,788 (0.02%, 0.00% to 0.15%)
5-9%	19,359 (0.00%)	16,436 (0.02%, 0.01% to 0.06%)
10-19%	12,072 (0.00%)	10,136 (0.02%, 0.00% to 0.08%)
20-29%	7,611 (0.00%)	6,275 (0.03%, 0.01% to 0.13%)
≥30%	2,038 (0.00%)	1,650 (0.00%)
Chi-square (p-value)	.	0.78 (p=0.941)
Low-lying vegetation		
0-4%	29,840 (0.00%)	25,102 (0.02%, 0.01% to 0.05%)
5-9%	15,366 (0.00%)	12,850 (0.02%, 0.01% to 0.07%)
≥10%	1,580 (0.00%)	1,333 (0.00%)
Chi-square (p-value)	.	0.33 (p=0.848)
95%CI: 95% Confidence Interval		

© 2019 Astell-Burt T et al. *JAMA Network Open.*

eTable 3: Cross-tabulation of missing self-rated fair or poor general health data at baseline and follow-up

	Self-rated fair or poor general health	
	Prevalence	Incidence
Total (% missing responses)	46786 (2.58%)	41494 (1.81%)
	Subtotal (% affirmative responses, 95%CI)	
Gender		
Male	21,633 (2.16%, 1.97% to 2.36%)	19,205 (2.28%, 2.08% to 2.50%)
Female	25,153 (2.95%, 2.75% to 3.17%)	22,289 (1.41%, 1.27% to 1.58%)
Chi-square (p-value)	28.92 (p<0.001)	43.56 (p<0.001)
Age group		
45-54y	15,443 (2.10%, 1.88% to 2.34%)	13,935 (0.82%, 0.68% to 0.98%)
55-64y	16,604 (2.43%, 2.21% to 2.68%)	14,869 (1.33%, 1.16% to 1.53%)
65-74y	9,178 (2.72%, 2.41% to 3.08%)	8,072 (2.58%, 2.25% to 2.95%)
≥75y	5,561 (4.15%, 3.66% to 4.71%)	4,618 (5.05%, 4.45% to 5.72%)
Chi-square (p-value)	71.15 (p<0.001)	393.99 (p<0.001)
Annual household income		
$0-$19,999	5,573 (2.60%, 2.21% to 3.05%)	4,316 (3.38%, 2.88% to 3.97%)
$20,000-$29,999	3,261 (1.72%, 1.32% to 2.23%)	2,813 (2.56%, 2.04% to 3.21%)
$30,000-$39,999	3,114 (1.51%, 1.14% to 2.00%)	2,809 (1.96%, 1.51% to 2.54%)
$40,000-$49,999	3,190 (1.19%, 0.87% to 1.63%)	2,893 (1.69%, 1.28% to 2.23%)
$50,000-$69,999	5,347 (0.92%, 0.69% to 1.21%)	4,929 (1.48%, 1.18% to 1.86%)
$70,000+	17,611 (0.61%, 0.50% to 0.73%)	16,647 (1.11%, 0.96% to 1.28%)
Not stated	8,690 (8.83%, 8.25% to 9.44%)	7,087 (2.46%, 2.12% to 2.84%)
Chi-square (p-value)	1,700.00 (p<0.001)	135.29 (p<0.001)
Highest educational qualification		
None	2,927 (4.85%, 4.13% to 5.69%)	2,246 (3.03%, 2.39% to 3.82%)
School	8,054 (3.23%, 2.86% to 3.64%)	6,904 (2.06%, 1.75% to 2.42%)
High school	4,419 (2.76%, 2.32% to 3.29%)	3,833 (2.01%, 1.61% to 2.50%)
Trade	4,170 (2.37%, 1.95% to 2.88%)	3,593 (2.28%, 1.84% to 2.83%)
Certificate/Diploma	10,366 (2.05%, 1.80% to 2.35%)	9,391 (1.61%, 1.37% to 1.88%)
University	16,398 (2.06%, 1.85% to 2.28%)	15,170 (1.44%, 1.27% to 1.65%)
Not stated	452 (7.96%, 5.80% to 10.85%)	357 (3.92%, 2.33% to 6.52%)
Chi-square (p-value)	156.07 (p<0.001)	48.90 (p<0.001)
Economic status		
Employed	26,040 (2.14%, 1.97% to 2.33%)	23,964 (1.19%, 1.06% to 1.33%)
Retired	16,762 (2.98%, 2.73% to 3.25%)	14,465 (2.83%, 2.58% to 3.12%)
Unemployed	645 (4.50%, 3.14% to 6.40%)	479 (1.88%, 0.98% to 3.57%)
Unpaid work	663 (2.56%, 1.60% to 4.09%)	595 (1.51%, 0.79% to 2.88%)
Disabled	566 (4.77%, 3.29% to 6.87%)	215 (2.79%, 1.26% to 6.08%)
Homemaker	1,541 (2.79%, 2.08% to 3.74%)	1,328 (1.58%, 1.03% to 2.41%)
Other (e.g. study)	569 (6.33%, 4.60% to 8.65%)	448 (2.90%, 1.69% to 4.94%)
Chi-square (p-value)	82.46 (p<0.001)	141.87 (p<0.001)

© 2019 Astell-Burt T et al. *JAMA Network Open.*

Couple status		
Not in a couple	10,236 (3.52%, 3.18% to 3.89%)	8,587 (2.24%, 1.94% to 2.57%)
In a couple	36,550 (2.32%, 2.17% to 2.48%)	32,907 (1.70%, 1.57% to 1.85%)
Chi-square (p-value)	45.30 (p<0.001)	10.78 (p=0.001)
Total green space		
0-4%	741 (2.56%, 1.64% to 3.99%)	628 (2.71%, 1.69% to 4.31%)
5-9%	11,056 (2.91%, 2.61% to 3.24%)	9,689 (1.88%, 1.63% to 2.17%)
10-19%	12,455 (2.83%, 2.56% to 3.14%)	10,953 (2.16%, 1.91% to 2.45%)
20-29%	12,712 (2.27%, 2.03% to 2.55%)	11,278 (1.70%, 1.48% to 1.96%)
≥30%	9,822 (2.30%, 2.02% to 2.62%)	8,946 (1.40%, 1.17% to 1.66%)
Chi-square (p-value)	15.83 (p=0.003)	20.06 (p<0.001)
Tree canopy		
0-9%	4,972 (3.04%, 2.59% to 3.55%)	4,135 (1.91%, 1.53% to 2.38%)
10-19%	18,041 (2.93%, 2.69% to 3.18%)	15,617 (1.97%, 1.77% to 2.20%)
20-29%	11,816 (2.46%, 2.20% to 2.76%)	10,684 (1.93%, 1.68% to 2.21%)
≥30%	11,957 (2.00%, 1.76% to 2.27%)	11,058 (1.45%, 1.24% to 1.69%)
Chi-square (p-value)	29.42 (p<0.001)	11.55 (p=0.009)
Grass		
0-4%	5,706 (2.54%, 2.16% to 2.98%)	5,212 (1.40%, 1.11% to 1.76%)
5-9%	19,359 (2.55%, 2.34% to 2.78%)	17,466 (1.85%, 1.66% to 2.06%)
10-19%	12,072 (2.44%, 2.18% to 2.73%)	10,592 (1.95%, 1.71% to 2.24%)
20-29%	7,611 (2.92%, 2.56% to 3.32%)	6,491 (1.86%, 1.56% to 2.22%)
≥30%	2,038 (2.65%, 2.03% to 3.44%)	1,733 (1.67%, 1.17% to 2.40%)
Chi-square (p-value)	4.56 (p=0.335)	6.57 (p=0.160)
Low-lying vegetation		
0-4%	29,840 (2.55%, 2.37% to 2.73%)	26,538 (1.88%, 1.73% to 2.05%)
5-9%	15,366 (2.66%, 2.41% to 2.92%)	13,568 (1.72%, 1.52% to 1.96%)
≥10%	1,580 (2.59%, 1.92% to 3.51%)	1,388 (1.37%, 0.87% to 2.14%)
Chi-square (p-value)	0.47 (p=0.789)	2.88 (p=0.237)
95%CI: 95% Confidence Interval		

© 2019 Astell-Burt T et al. *JAMA Network Open.*

eTable 4: Associations between total green space, green space type, and prevalent Kessler 10 psychological distress adjusted for confounding in multilevel logistic regressions estimated with Markov Chain Monte Carlo (MCMC)

	10-item Kessler Psychological Distress Scale (prevalence)			
	Model 1	Model 2	Model 3	Model 4
	Total green space	Tree canopy	Grass	Low-lying vegetation
	Odds Ratio (95% Confidence Interval) [p-value]			
Total green space (ref: 0-4%)				
5-9%	0.82 (0.58 to 1.18) [p=0.12]			
10-19%	0.80 (0.56 to 1.17) [p=0.11]			
20-29%	0.79 (0.55 to 1.15) [p=0.09]			
≥30%	0.69 (0.47 to 1.02) [p=0.03]			
Tree canopy (ref: 0-9%)				
10-19%		0.81 (0.70 to 0.94) [p=0.003]		
20-29%		0.73 (0.61 to 0.87) [p=0.001]		
≥30%		0.61 (0.50 to 0.74) [p<0.001]		
Grass (ref: 0-4%)				
5-9%			1.13 (0.93 to 1.35) [p=0.12]	
10-19%			1.38 (1.11 to 1.70) [p=0.001]	
20-29%			1.27 (0.99 to 1.60) [p=0.03]	
≥30%			1.71 (1.25 to 2.28) [p<0.001]	
Low-lying vegetation (0-4%)				
5-9%				0.97 (0.86 to 1.08) [p=0.26]
≥10%				0.97 (0.72 to 1.26) [p=0.39]
Age group (ref: 45-54y)				
55-64y	0.55 (0.49 to 0.61) [p<0.001]	0.55 (0.49 to 0.62) [p<0.001]	0.55 (0.49 to 0.62) [p<0.001]	0.55 (0.49 to 0.62) [p<0.001]

© 2019 Astell-Burt T et al. *JAMA Network Open.*

65-74y	0.27 (0.22 to 0.32) [p<0.001]	0.27 (0.22 to 0.32) [p<0.001]	0.27 (0.22 to 0.32) [p<0.001]	0.27 (0.22 to 0.32) [p<0.001]
≥75y	0.22 (0.17 to 0.28) [p<0.001]	0.23 (0.18 to 0.28) [p<0.001]	0.23 (0.18 to 0.29) [p<0.001]	0.22 (0.18 to 0.28) [p<0.001]
Gender (ref: Male)				
Female	1.06 (0.95 to 1.17) [p=0.14]	1.06 (0.96 to 1.18) [p=0.12]	1.06 (0.95 to 1.17) [p=0.15]	1.06 (0.95 to 1.17) [p=0.15]
Annual household income (ref: $0-$19,999)				
$20,000-$29,999	0.77 (0.63 to 0.95) [p=0.007]	0.77 (0.63 to 0.94) [p=0.004]	0.77 (0.62 to 0.94) [p=0.005]	0.77 (0.62 to 0.93) [p=0.004]
$30,000-$39,999	0.72 (0.58 to 0.89) [p=0.001]	0.72 (0.58 to 0.88) [p=0.001]	0.72 (0.58 to 0.89) [p=0.001]	0.72 (0.58 to 0.88) [p=0.001]
$40,000-$49,999	0.54 (0.42 to 0.67) [p<0.001]	0.54 (0.43 to 0.67) [p<0.001]	0.53 (0.42 to 0.66) [p<0.001]	0.53 (0.42 to 0.66) [p<0.001]
$50,000-$69,999	0.49 (0.40 to 0.60) [p<0.001]	0.49 (0.40 to 0.59) [p<0.001]	0.49 (0.40 to 0.60) [p<0.001]	0.49 (0.40 to 0.59) [p<0.001]
$70,000+	0.41 (0.34 to 0.49) [p<0.001]	0.41 (0.35 to 0.49) [p<0.001]	0.41 (0.34 to 0.49) [p<0.001]	0.41 (0.34 to 0.49) [p<0.001]
Not stated	0.56 (0.47 to 0.66) [p<0.001]	0.56 (0.47 to 0.66) [p<0.001]	0.56 (0.47 to 0.66) [p<0.001]	0.55 (0.47 to 0.65) [p<0.001]
Highest educational qualification (ref: None)				
School	0.69 (0.57 to 0.84) [p<0.001]	0.71 (0.58 to 0.86) [p<0.001]	0.70 (0.57 to 0.84) [p<0.001]	0.69 (0.57 to 0.84) [p<0.001]
High school	0.69 (0.55 to 0.85) [p<0.001]	0.71 (0.56 to 0.88) [p=0.001]	0.70 (0.56 to 0.87) [p<0.001]	0.69 (0.55 to 0.86) [p<0.001]
Trade	0.69 (0.55 to 0.86) [p=0.001]	0.71 (0.56 to 0.88) [p=0.001]	0.70 (0.56 to 0.88) [p=0.001]	0.69 (0.55 to 0.86) [p=0.001]
Certificate/Diploma	0.64 (0.53 to 0.78) [p<0.001]	0.66 (0.54 to 0.80) [p<0.001]	0.65 (0.53 to 0.79) [p<0.001]	0.64 (0.53 to 0.77) [p<0.001]
University	0.60 (0.49 to 0.73) [p<0.001]	0.62 (0.51 to 0.76) [p<0.001]	0.62 (0.50 to 0.75) [p<0.001]	0.60 (0.49 to 0.73) [p<0.001]
Not stated	1.16 (0.74 to 1.72) [p=0.28]	1.19 (0.75 to 1.76) [p=0.25]	1.17 (0.73 to 1.74) [p=0.27]	1.16 (0.73 to 1.72) [p=0.27]
Economic status (ref: Employed)				
Retired	1.18 (1.01 to 1.38) [p=0.019]	1.18 (1.02 to 1.37) [p=0.016]	1.18 (1.01 to 1.36) [p=0.021]	1.18 (1.01 to 1.36) [p=0.021]
Unemployed	3.17 (2.44 to 4.02) [p<0.001]	3.19 (2.46 to 4.07) [p<0.001]	3.18 (2.45 to 4.04) [p<0.001]	3.18 (2.45 to 4.03) [p<0.001]
Unpaid work	1.72 (1.17 to 2.38) [p=0.003]	1.72 (1.18 to 2.40) [p=0.003]	1.71 (1.16 to 2.37) [p=0.003]	1.71 (1.18 to 2.36) [p=0.003]
Disabled	6.80 (5.33 to 8.52) [p<0.001]	6.73 (5.28 to 8.44) [p<0.001]	6.89 (5.41 to 8.66) [p<0.001]	6.84 (5.37 to 8.57) [p<0.001]
Homemaker	1.74 (1.37 to 2.16) [p<0.001]	1.74 (1.36 to 2.17) [p<0.001]	1.74 (1.37 to 2.17) [p<0.001]	1.74 (1.37 to 2.16) [p<0.001]
Other (e.g. study)	2.21 (1.53 to 3.07) [p<0.001]	2.22 (1.53 to 3.08) [p<0.001]	2.22 (1.52 to 3.05) [p<0.001]	2.21 (1.52 to 3.05) [p<0.001]

© 2019 Astell-Burt T et al. *JAMA Network Open.*

Couple status (ref: Not in a couple)				
In a couple	0.60 (0.54 to 0.67) [p<0.001]	0.61 (0.54 to 0.68) [p<0.001]	0.59 (0.53 to 0.66) [p<0.001]	0.60 (0.54 to 0.67) [p<0.001]

© 2019 Astell-Burt T et al. *JAMA Network Open.*

eTable 5: Associations between total green space, green space type, and incident Kessler 10 psychological distress adjusted for confounding in multilevel logistic regressions estimated with Markov Chain Monte Carlo (MCMC)

	10-item Kessler Psychological Distress Scale (incidence)			
	Model 5	Model 6	Model 7	Model 8
	Total green space	Tree canopy	Grass	Low-lying vegetation
	Odds Ratio (95% Confidence Interval) [p-value]			
Total green space (ref: 0-4%)				
5-9%	0.54 (0.36 to 0.80) [p=0.003]			
10-19%	0.53 (0.35 to 0.79) [p=0.003]			
20-29%	0.50 (0.33 to 0.75) [p<0.001]			
≥30%	0.46 (0.29 to 0.69) [p<0.001]			
Tree canopy (ref: 0-9%)				
10-19%		0.92 (0.75 to 1.11) [p=0.18]		
20-29%		0.76 (0.60 to 0.95) [p=0.009]		
≥30%		0.69 (0.54 to 0.88) [p=0.002]		
Grass (ref: 0-4%)				
5-9%			0.89 (0.71 to 1.11) [p=0.14]	
10-19%			1.05 (0.81 to 1.33) [p=0.38]	
20-29%			1.07 (0.81 to 1.39) [p=0.35]	
≥30%			0.90 (0.59 to 1.30) [p=0.27]	
Low-lying vegetation (0-4%)				
5-9%				1.06 (0.92 to 1.22) [p=0.21]
≥10%				1.11 (0.77 to 1.51) [p=0.30]
Age group (ref: 45-54y)				
55-64y	0.59 (0.51 to 0.69) [p<0.001]	0.60 (0.51 to 0.70) [p<0.001]	0.59 (0.51 to 0.69) [p<0.001]	0.59 (0.51 to 0.69) [p<0.001]

© 2019 Astell-Burt T et al. *JAMA Network Open.*

65-74y	0.50 (0.39 to 0.63) [p<0.001]	0.51 (0.40 to 0.64) [p<0.001]	0.51 (0.40 to 0.63) [p<0.001]	0.50 (0.40 to 0.63) [p<0.001]
≥75y	0.62 (0.46 to 0.81) [p<0.001]	0.64 (0.48 to 0.84) [p=0.001]	0.63 (0.47 to 0.82) [p=0.001]	0.63 (0.47 to 0.81) [p<0.001]
Gender (ref: Male)				
Female	1.14 (0.99 to 1.30) [p=0.033]	1.15 (1.00 to 1.31) [p=0.025]	1.14 (0.99 to 1.30) [p=0.033]	1.14 (0.99 to 1.30) [p=0.033]
Annual household income (ref: $0-$19,999)				
$20,000-$29,999	0.97 (0.73 to 1.26) [p=0.39]	0.98 (0.74 to 1.27) [p=0.41]	0.96 (0.72 to 1.26) [p=0.35]	0.97 (0.73 to 1.26) [p=0.373]
$30,000-$39,999	0.74 (0.54 to 0.99) [p=0.020]	0.76 (0.55 to 1.01) [p=0.029]	0.74 (0.54 to 0.98) [p=0.020]	0.74 (0.54 to 0.99) [p=0.020]
$40,000-$49,999	0.87 (0.65 to 1.14) [p=0.146]	0.88 (0.66 to 1.16) [p=0.18]	0.86 (0.64 to 1.13) [p=0.127]	0.86 (0.64 to 1.13) [p=0.139]
$50,000-$69,999	0.65 (0.49 to 0.84) [p=0.001]	0.67 (0.51 to 0.87) [p=0.001]	0.65 (0.49 to 0.84) [p=0.001]	0.65 (0.49 to 0.85) [p=0.001]
$70,000+	0.52 (0.40 to 0.66) [p<0.001]	0.54 (0.42 to 0.69) [p<0.001]	0.52 (0.41 to 0.66) [p<0.001]	0.52 (0.41 to 0.66) [p<0.001]
Not stated	0.78 (0.62 to 0.97) [p=0.015]	0.80 (0.63 to 1.00) [p=0.023]	0.78 (0.61 to 0.97) [p=0.014]	0.78 (0.62 to 0.98) [p=0.015]
Highest educational qualification (ref: None)				
School	0.65 (0.51 to 0.84) [p=0.001]	0.68 (0.52 to 0.86) [p=0.001]	0.66 (0.51 to 0.84) [p=0.001]	0.65 (0.50 to 0.84) [p=0.001]
High school	0.56 (0.41 to 0.75) [p<0.001]	0.59 (0.43 to 0.78) [p<0.001]	0.57 (0.42 to 0.75) [p<0.001]	0.56 (0.42 to 0.75) [p<0.001]
Trade	0.69 (0.51 to 0.91) [p=0.005]	0.71 (0.52 to 0.93) [p=0.008]	0.69 (0.51 to 0.91) [p=0.004]	0.68 (0.51 to 0.92) [p=0.006]
Certificate/Diploma	0.63 (0.49 to 0.80) [p<0.001]	0.66 (0.51 to 0.83) [p<0.001]	0.63 (0.49 to 0.81) [p<0.001]	0.62 (0.48 to 0.81) [p<0.001]
University	0.49 (0.38 to 0.63) [p<0.001]	0.52 (0.41 to 0.67) [p<0.001]	0.50 (0.39 to 0.64) [p<0.001]	0.50 (0.38 to 0.64) [p<0.001]
Not stated	0.78 (0.37 to 1.37) [p=0.18]	0.82 (0.39 to 1.44) [p=0.23]	0.80 (0.38 to 1.41) [p=0.20]	0.79 (0.38 to 1.40) [p=0.19]
Economic status (ref: Employed)				
Retired	0.97 (0.79 to 1.19) [p=0.38]	0.98 (0.80 to 1.19) [p=0.40]	0.97 (0.79 to 1.18) [p=0.36]	0.97 (0.79 to 1.17) [p=0.36]
Unemployed	2.15 (1.43 to 3.08) [p<0.001]	2.17 (1.43 to 3.11) [p<0.001]	2.15 (1.40 to 3.07) [p<0.001]	2.16 (1.41 to 3.10) [p<0.001]
Unpaid work	0.90 (0.47 to 1.47) [p=0.31]	0.90 (0.47 to 1.50) [p=0.33]	0.90 (0.47 to 1.48) [p=0.33]	0.89 (0.46 to 1.47) [p=0.31]
Disabled	2.59 (1.63 to 3.82) [p<0.001]	2.64 (1.65 to 3.93) [p<0.001]	2.62 (1.63 to 3.87) [p<0.001]	2.64 (1.66 to 3.92) [p<0.001]
Homemaker	1.09 (0.76 to 1.50) [p=0.33]	1.11 (0.77 to 1.51) [p=0.31]	1.10 (0.77 to 1.49) [p=0.32]	1.09 (0.76 to 1.49) [p=0.33]
Other (e.g. study)	1.68 (0.97 to 2.62) [p=0.033]	1.70 (0.98 to 2.66) [p=0.028]	1.67 (0.95 to 2.60) [p=0.035]	1.67 (0.95 to 2.63) [p=0.038]
Couple status (ref: Not in a couple)				

© 2019 Astell-Burt T et al. *JAMA Network Open.*

In a couple	0.88 (0.75 to 1.02) [p=0.045]	0.87 (0.75 to 1.01) [p=0.035]	0.86 (0.74 to 0.99) [p=0.020]	0.86 (0.73 to 1.00) [p=0.025]

© 2019 Astell-Burt T et al. *JAMA Network Open.*

eTable 6: Associations between total green space, green space type, and prevalent self-reported physician-diagnosed depression or anxiety adjusted for confounding in multilevel logistic regressions estimated with Markov Chain Monte Carlo (MCMC)

	Self-reported physician-diagnosed depression or anxiety (prevalence)			
	Model 9	Model 10	Model 11	Model 12
	Total green space	Tree canopy	Grass	Low-lying vegetation
	Odds Ratio (95% Confidence Interval) [p-value]			
Total green space (ref: 0-4%)				
5-9%	0.94 (0.75 to 1.16) [p=0.27]			
10-19%	0.92 (0.73 to 1.14) [p=0.21]			
20-29%	0.95 (0.75 to 1.18) [p=0.30]			
≥30%	0.93 (0.74 to 1.16) [p=0.26]			
Tree canopy (ref: 0-9%)				
10-19%		1.01 (0.92 to 1.10) [p=0.42]		
20-29%		1.00 (0.90 to 1.11) [p=0.48]		
≥30%		0.97 (0.86 to 1.09) [p=0.29]		
Grass (ref: 0-4%)				
5-9%			0.98 (0.90 to 1.07) [p=0.33]	
10-19%			0.98 (0.87 to 1.09) [p=0.36]	
20-29%			1.01 (0.89 to 1.14) [p=0.43]	
≥30%			1.12 (0.94 to 1.32) [p=0.10]	
Low-lying vegetation (0-4%)				
5-9%				1.04 (0.98 to 1.11) [p=0.08]
≥10%				0.97 (0.83 to 1.12) [p=0.36]
Age group (ref: 45-54y)				
55-64y	0.81 (0.76 to 0.86) [p<0.001]	0.81 (0.76 to 0.86) [p<0.001]	0.81 (0.76 to 0.86) [p<0.001]	0.81 (0.76 to 0.86) [p<0.001]

© 2019 Astell-Burt T et al. *JAMA Network Open.*

65-74y	0.55 (0.50 to 0.61) [p<0.001]	0.55 (0.50 to 0.60) [p<0.001]	0.55 (0.50 to 0.60) [p<0.001]	0.55 (0.50 to 0.60) [p<0.001]
≥75y	0.36 (0.31 to 0.40) [p<0.001]	0.36 (0.32 to 0.40) [p<0.001]	0.36 (0.32 to 0.40) [p<0.001]	0.36 (0.32 to 0.40) [p<0.001]
Gender (ref: Male)				
Female	1.75 (1.66 to 1.86) [p<0.001]	1.75 (1.65 to 1.85) [p<0.001]	1.75 (1.66 to 1.85) [p<0.001]	1.75 (1.65 to 1.85) [p<0.001]
Annual household income (ref: $0-$19,999)				
$20,000-$29,999	0.93 (0.83 to 1.05) [p=0.13]	0.94 (0.83 to 1.05) [p=0.12]	0.94 (0.83 to 1.05) [p=0.13]	0.94 (0.83 to 1.05) [p=0.13]
$30,000-$39,999	0.92 (0.81 to 1.03) [p=0.08]	0.92 (0.81 to 1.03) [p=0.07]	0.92 (0.81 to 1.04) [p=0.08]	0.92 (0.81 to 1.03) [p=0.08]
$40,000-$49,999	0.82 (0.73 to 0.93) [p=0.001]	0.82 (0.73 to 0.93) [p=0.001]	0.82 (0.73 to 0.93) [p=0.001]	0.83 (0.73 to 0.93) [p=0.002]
$50,000-$69,999	0.84 (0.75 to 0.94) [p=0.001]	0.84 (0.75 to 0.94) [p=0.001]	0.84 (0.75 to 0.94) [p=0.002]	0.84 (0.75 to 0.94) [p=0.001]
$70,000+	0.79 (0.71 to 0.87) [p<0.001]	0.79 (0.71 to 0.87) [p<0.001]	0.79 (0.71 to 0.88) [p<0.001]	0.79 (0.71 to 0.88) [p<0.001]
Not stated	0.69 (0.63 to 0.76) [p<0.001]	0.69 (0.63 to 0.76) [p<0.001]	0.69 (0.63 to 0.76) [p<0.001]	0.69 (0.63 to 0.76) [p<0.001]
Highest educational qualification (ref: None)				
School	0.85 (0.75 to 0.95) [p=0.002]	0.85 (0.76 to 0.94) [p=0.001]	0.85 (0.76 to 0.95) [p=0.003]	0.84 (0.75 to 0.94) [p=0.003]
High school	0.87 (0.76 to 0.99) [p=0.016]	0.87 (0.76 to 0.98) [p=0.014]	0.87 (0.77 to 0.99) [p=0.019]	0.87 (0.76 to 0.99) [p=0.015]
Trade	0.83 (0.72 to 0.95) [p=0.004]	0.83 (0.73 to 0.95) [p=0.003]	0.84 (0.73 to 0.95) [p=0.005]	0.83 (0.72 to 0.95) [p=0.004]
Certificate/Diploma	0.98 (0.87 to 1.10) [p=0.34]	0.98 (0.87 to 1.09) [p=0.35]	0.98 (0.88 to 1.10) [p=0.37]	0.97 (0.87 to 1.09) [p=0.31]
University	0.94 (0.84 to 1.06) [p=0.16]	0.95 (0.84 to 1.06) [p=0.17]	0.95 (0.85 to 1.07) [p=0.19]	0.94 (0.84 to 1.06) [p=0.15]
Not stated	1.00 (0.75 to 1.30) [p=0.48]	1.00 (0.76 to 1.29) [p=0.48]	1.00 (0.75 to 1.30) [p=0.48]	0.99 (0.75 to 1.29) [p=0.46]
Economic status (ref: Employed)				
Retired	1.30 (1.20 to 1.40) [p<0.001]	1.30 (1.20 to 1.40) [p<0.001]	1.30 (1.20 to 1.40) [p<0.001]	1.29 (1.20 to 1.40) [p<0.001]
Unemployed	1.85 (1.53 to 2.20) [p<0.001]	1.85 (1.54 to 2.21) [p<0.001]	1.85 (1.53 to 2.21) [p<0.001]	1.85 (1.54 to 2.21) [p<0.001]
Unpaid work	1.28 (1.03 to 1.55) [p=0.013]	1.28 (1.04 to 1.55) [p=0.010]	1.28 (1.03 to 1.55) [p=0.012]	1.28 (1.03 to 1.55) [p=0.013]
Disabled	4.46 (3.70 to 5.33) [p<0.001]	4.47 (3.71 to 5.33) [p<0.001]	4.46 (3.70 to 5.33) [p<0.001]	4.45 (3.70 to 5.30) [p<0.001]
Homemaker	1.24 (1.07 to 1.41) [p=0.002]	1.24 (1.07 to 1.42) [p=0.001]	1.24 (1.07 to 1.42) [p=0.001]	1.24 (1.07 to 1.41) [p=0.002]
Other (e.g. study)	1.47 (1.17 to 1.82) [p<0.001]	1.47 (1.18 to 1.81) [p<0.001]	1.47 (1.17 to 1.82) [p<0.001]	1.47 (1.17 to 1.81) [p=0.001]

© 2019 Astell-Burt T et al. *JAMA Network Open.*

Couple status (ref: Not in a couple)				
In a couple	0.63 (0.59 to 0.67) [p<0.001]	0.63 (0.59 to 0.67) [p<0.001]	0.63 (0.59 to 0.67) [p<0.001]	0.63 (0.59 to 0.67) [p<0.001]

© 2019 Astell-Burt T et al. *JAMA Network Open.*

eTable 7: Associations between total green space, green space type, and incident self-reported physician-diagnosed depression or anxiety adjusted for confounding in multilevel logistic regressions estimated with Markov Chain Monte Carlo (MCMC)

	Self-reported physician-diagnosed depression or anxiety (incidence)			
	Model 13	Model 14	Model 15	Model 16
	Total green space	Tree canopy	Grass	Low-lying vegetation
	Odds Ratio (95% Confidence Interval) [p-value]			
Total green space (ref: 0-4%)				
5-9%	1.37 (0.99 to 1.88) [p=0.027]			
10-19%	1.30 (0.94 to 1.78) [p=0.06]			
20-29%	1.42 (1.03 to 1.94) [p=0.014]			
≥30%	1.21 (0.87 to 1.67) [p=0.14]			
Tree canopy (ref: 0-9%)				
10-19%		0.97 (0.86 to 1.10) [p=0.67]		
20-29%		0.85 (0.74 to 0.98) [p=0.029]		
≥30%		0.86 (0.74 to 1.00) [p=0.05]		
Grass (ref: 0-4%)				
5-9%			0.96 (0.84 to 1.10) [p=0.28]	
10-19%			1.06 (0.91 to 1.24) [p=0.23]	
20-29%			1.08 (0.90 to 1.28) [p=0.22]	
≥30%			1.13 (0.88 to 1.43) [p=0.17]	
Low-lying vegetation (0-4%)				
5-9%				1.02 (0.94 to 1.12) [p=0.31]
≥10%				1.04 (0.83 to 1.28) [p=0.39]
Age group (ref: 45-54y)				
55-64y	0.67 (0.61 to 0.74) [p<0.001]	0.68 (0.61 to 0.74) [p<0.001]	0.68 (0.61 to 0.74) [p<0.001]	0.67 (0.61 to 0.74) [p<0.001]

© 2019 Astell-Burt T et al. *JAMA Network Open.*

65-74y	0.50 (0.43 to 0.58) [p<0.001]	0.50 (0.43 to 0.58) [p<0.001]	0.50 (0.43 to 0.58) [p<0.001]	0.50 (0.43 to 0.57) [p<0.001]
≥75y	0.49 (0.41 to 0.58) [p<0.001]	0.50 (0.42 to 0.59) [p<0.001]	0.50 (0.42 to 0.58) [p<0.001]	0.49 (0.41 to 0.58) [p<0.001]
Gender (ref: Male)				
Female	1.38 (1.27 to 1.50) [p<0.001]	1.38 (1.27 to 1.50) [p<0.001]	1.38 (1.27 to 1.50) [p<0.001]	1.38 (1.27 to 1.50) [p<0.001]
Annual household income (ref: $0-$19,999)				
$20,000-$29,999	1.00 (0.83 to 1.19) [p=0.47]	1.00 (0.83 to 1.20) [p=0.99]	1.00 (0.83 to 1.19) [p=0.48]	1.00 (0.83 to 1.19) [p=0.48]
$30,000-$39,999	0.97 (0.79 to 1.16) [p=0.355]	0.97 (0.80 to 1.17) [p=0.76]	0.97 (0.80 to 1.17) [p=0.37]	0.97 (0.80 to 1.16) [p=0.36]
$40,000-$49,999	0.87 (0.71 to 1.05) [p=0.07]	0.87 (0.72 to 1.06) [p=0.17]	0.87 (0.71 to 1.05) [p=0.08]	0.87 (0.71 to 1.05) [p=0.071]
$50,000-$69,999	1.04 (0.87 to 1.22) [p=0.36]	1.03 (0.87 to 1.22) [p=0.69]	1.04 (0.88 to 1.22) [p=0.341]	1.04 (0.87 to 1.22) [p=0.35]
$70,000+	0.85 (0.73 to 0.99) [p=0.021]	0.85 (0.73 to 1.00) [p=0.048]	0.86 (0.73 to 1.00) [p=0.023]	0.85 (0.73 to 0.99) [p=0.019]
Not stated	1.14 (0.99 to 1.31) [p=0.039]	1.14 (0.99 to 1.31) [p=0.08]	1.14 (0.99 to 1.31) [p=0.031]	1.14 (0.99 to 1.31) [p=0.038]
Highest educational qualification (ref: None)				
School	0.77 (0.65 to 0.91) [p=0.001]	0.77 (0.66 to 0.91) [p=0.002]	0.77 (0.65 to 0.91) [p=0.001]	0.77 (0.65 to 0.90) [p<0.001]
High school	0.68 (0.56 to 0.81) [p<0.001]	0.67 (0.56 to 0.81) [p<0.001]	0.68 (0.55 to 0.81) [p<0.001]	0.67 (0.55 to 0.80) [p<0.001]
Trade	0.77 (0.64 to 0.93) [p=0.003]	0.77 (0.64 to 0.93) [p=0.007]	0.77 (0.64 to 0.93) [p=0.003]	0.77 (0.63 to 0.92) [p=0.002]
Certificate/Diploma	0.75 (0.64 to 0.88) [p<0.001]	0.76 (0.64 to 0.89) [p=0.001]	0.75 (0.64 to 0.88) [p<0.001]	0.75 (0.63 to 0.87) [p<0.001]
University	0.67 (0.57 to 0.79) [p<0.001]	0.67 (0.57 to 0.79) [p<0.001]	0.67 (0.57 to 0.79) [p<0.001]	0.67 (0.56 to 0.78) [p<0.001]
Not stated	0.86 (0.56 to 1.23) [p=0.20]	0.85 (0.58 to 1.25) [p=0.41]	0.85 (0.55 to 1.22) [p=0.18]	0.85 (0.56 to 1.23) [p=0.18]
Economic status (ref: Employed)				
Retired	1.18 (1.05 to 1.33) [p=0.003]	1.19 (1.05 to 1.33) [p=0.005]	1.18 (1.05 to 1.33) [p=0.003]	1.19 (1.05 to 1.33) [p=0.003]
Unemployed	1.46 (1.07 to 1.92) [p=0.008]	1.45 (1.08 to 1.94) [p=0.012]	1.47 (1.07 to 1.93) [p=0.009]	1.47 (1.07 to 1.94) [p=0.010]
Unpaid work	1.35 (0.99 to 1.77) [p=0.028]	1.35 (1.01 to 1.81) [p=0.042]	1.36 (1.00 to 1.79) [p=0.025]	1.36 (1.00 to 1.78) [p=0.024]
Disabled	2.29 (1.62 to 3.09) [p<0.001]	2.26 (1.63 to 3.12) [p<0.001]	2.28 (1.61 to 3.11) [p<0.001]	2.30 (1.62 to 3.10) [p<0.001]
Homemaker	1.05 (0.84 to 1.28) [p=0.35]	1.05 (0.85 to 1.29) [p=0.67]	1.05 (0.84 to 1.28) [p=0.35]	1.05 (0.84 to 1.28) [p=0.35]
Other (e.g. study)	1.48 (1.06 to 1.99) [p=0.011]	1.48 (1.09 to 2.01) [p=0.013]	1.48 (1.07 to 1.98) [p=0.011]	1.49 (1.07 to 1.98) [p=0.009]

© 2019 Astell-Burt T et al. *JAMA Network Open.*

Couple status (ref: Not in a couple)				
In a couple	0.84 (0.76 to 0.92) [p<0.001]	0.84 (0.77 to 0.93) [p<0.001]	0.83 (0.76 to 0.91) [p<0.001]	0.84 (0.76 to 0.92) [p<0.001]

© 2019 Astell-Burt T et al. *JAMA Network Open.*

eTable 8: Associations between total green space, green space type, and prevalent missing self-rated fair or poor general health adjusted for confounding in multilevel logistic regressions estimated with Markov Chain Monte Carlo (MCMC)

	Self-rated fair-to-poor general health (prevalence)			
	Model 17	Model 18	Model 19	Model 20
	Total green space	Tree canopy	Grass	Low-lying vegetation
	Odds Ratio (95% Confidence Interval) [p-value]			
Total green space (ref: 0-4%)				
5-9%	0.76 (0.57 to 1.00) [p=0.023]			
10-19%	0.75 (0.56 to 0.97) [p=0.015]			
20-29%	0.74 (0.55 to 0.97) [p=0.014]			
≥30%	0.63 (0.46 to 0.83) [p=0.001]			
Tree canopy (ref: 0-9%)				
10-19%		0.94 (0.84 to 1.05) [p=0.17]		
20-29%		0.77 (0.67 to 0.88) [p<0.001]		
≥30%		0.66 (0.56 to 0.78) [p<0.001]		
Grass (ref: 0-4%)				
5-9%			1.05 (0.91 to 1.20) [p=0.28]	
10-19%			1.16 (0.96 to 1.36) [p=0.06]	
20-29%			1.24 (1.00 to 1.51) [p=0.026]	
≥30%			1.40 (1.07 to 1.78) [p=0.006]	
Low-lying vegetation (0-4%)				
5-9%				0.95 (0.87 to 1.04) [p=0.14]
≥10%				1.11 (0.91 to 1.35) [p=0.15]
Age group (ref: 45-54y)				
55-64y	0.80 (0.73 to 0.88) [p<0.001]	0.80 (0.73 to 0.88) [p<0.001]	0.80 (0.73 to 0.88) [p<0.001]	0.80 (0.73 to 0.88) [p<0.001]

© 2019 Astell-Burt T et al. *JAMA Network Open.*

65-74y	0.72 (0.63 to 0.81) [p<0.001]	0.72 (0.64 to 0.82) [p<0.001]	0.72 (0.63 to 0.81) [p<0.001]	0.72 (0.63 to 0.81) [p<0.001]
≥75y	0.87 (0.76 to 1.00) [p=0.023]	0.88 (0.76 to 1.00) [p=0.029]	0.87 (0.76 to 1.00) [p=0.024]	0.87 (0.76 to 1.00) [p=0.025]
Gender (ref: Male)				
Female	0.79 (0.74 to 0.85) [p<0.001]	0.79 (0.74 to 0.85) [p<0.001]	0.79 (0.74 to 0.85) [p<0.001]	0.79 (0.74 to 0.85) [p<0.001]
Annual household income (ref: $0-$19,999)				
$20,000-$29,999	0.74 (0.65 to 0.84) [p<0.001]	0.74 (0.65 to 0.84) [p<0.001]	0.74 (0.65 to 0.84) [p<0.001]	0.74 (0.64 to 0.84) [p<0.001]
$30,000-$39,999	0.56 (0.48 to 0.65) [p<0.001]	0.56 (0.48 to 0.65) [p<0.001]	0.56 (0.48 to 0.64) [p<0.001]	0.56 (0.48 to 0.64) [p<0.001]
$40,000-$49,999	0.58 (0.49 to 0.68) [p<0.001]	0.58 (0.50 to 0.68) [p<0.001]	0.58 (0.49 to 0.67) [p<0.001]	0.58 (0.50 to 0.67) [p<0.001]
$50,000-$69,999	0.53 (0.45 to 0.60) [p<0.001]	0.53 (0.46 to 0.61) [p<0.001]	0.53 (0.46 to 0.60) [p<0.001]	0.53 (0.46 to 0.60) [p<0.001]
$70,000+	0.44 (0.39 to 0.50) [p<0.001]	0.44 (0.39 to 0.51) [p<0.001]	0.44 (0.39 to 0.50) [p<0.001]	0.44 (0.39 to 0.50) [p<0.001]
Not stated	0.70 (0.63 to 0.78) [p<0.001]	0.70 (0.63 to 0.78) [p<0.001]	0.70 (0.63 to 0.78) [p<0.001]	0.70 (0.63 to 0.78) [p<0.001]
Highest educational qualification (ref: None)				
School	0.73 (0.65 to 0.83) [p<0.001]	0.74 (0.66 to 0.84) [p<0.001]	0.74 (0.65 to 0.84) [p<0.001]	0.74 (0.65 to 0.83) [p<0.001]
High school	0.80 (0.69 to 0.93) [p=0.001]	0.82 (0.70 to 0.95) [p=0.005]	0.81 (0.70 to 0.94) [p=0.003]	0.81 (0.70 to 0.93) [p=0.001]
Trade	0.72 (0.62 to 0.84) [p<0.001]	0.74 (0.64 to 0.85) [p<0.001]	0.73 (0.63 to 0.84) [p<0.001]	0.73 (0.63 to 0.84) [p<0.001]
Certificate/Diploma	0.60 (0.52 to 0.68) [p<0.001]	0.61 (0.54 to 0.69) [p<0.001]	0.60 (0.53 to 0.69) [p<0.001]	0.60 (0.52 to 0.68) [p<0.001]
University	0.55 (0.48 to 0.63) [p<0.001]	0.56 (0.49 to 0.64) [p<0.001]	0.56 (0.48 to 0.64) [p<0.001]	0.55 (0.48 to 0.63) [p<0.001]
Not stated	0.85 (0.61 to 1.14) [p=0.13]	0.86 (0.62 to 1.16) [p=0.16]	0.85 (0.61 to 1.14) [p=0.14]	0.85 (0.61 to 1.14) [p=0.13]
Economic status (ref: Employed)				
Retired	1.51 (1.35 to 1.67) [p<0.001]	1.51 (1.35 to 1.68) [p<0.001]	1.51 (1.36 to 1.67) [p<0.001]	1.51 (1.36 to 1.68) [p<0.001]
Unemployed	2.66 (2.14 to 3.26) [p<0.001]	2.67 (2.15 to 3.27) [p<0.001]	2.67 (2.15 to 3.25) [p<0.001]	2.66 (2.14 to 3.26) [p<0.001]
Unpaid work	1.19 (0.86 to 1.58) [p=0.15]	1.20 (0.88 to 1.58) [p=0.13]	1.20 (0.87 to 1.58) [p=0.13]	1.20 (0.87 to 1.59) [p=0.14]
Disabled	11.87 (9.71 to 14.33) [p<0.001]	11.86 (9.70 to 14.38) [p<0.001]	11.97 (9.77 to 14.49) [p<0.001]	11.98 (9.79 to 14.48) [p<0.001]
Homemaker	1.70 (1.41 to 2.01) [p<0.001]	1.71 (1.42 to 2.03) [p<0.001]	1.70 (1.42 to 2.02) [p<0.001]	1.70 (1.41 to 2.03) [p<0.001]
Other (e.g. study)	2.03 (1.56 to 2.57) [p<0.001]	2.04 (1.57 to 2.60) [p<0.001]	2.04 (1.57 to 2.60) [p<0.001]	2.04 (1.57 to 2.58) [p<0.001]

© 2019 Astell-Burt T et al. *JAMA Network Open.*

Couple status (ref: Not in a couple)				
In a couple	0.74 (0.69 to 0.80) [p<0.001]	0.75 (0.69 to 0.81) [p<0.001]	0.74 (0.68 to 0.80) [p<0.001]	0.74 (0.68 to 0.80) [p<0.001]

© 2019 Astell-Burt T et al. *JAMA Network Open.*

eTable 9: Associations between total green space, green space type, and incident missing self-rated fair or poor general health adjusted for confounding in multilevel logistic regressions estimated with Markov Chain Monte Carlo (MCMC)

	Self-rated fair-to-poor general health (incidence)			
	Model 21	Model 22	Model 23	Model 24
	Total green space	Tree canopy	Grass	Low-lying vegetation
	Odds Ratio (95% Confidence Interval) [p-value]			
Total green space (ref: 0-4%)				
5-9%	1.55 (1.01 to 2.34) [p=0.023]			
10-19%	1.60 (1.03 to 2.43) [p=0.018]			
20-29%	1.52 (0.97 to 2.33) [p=0.034]			
≥30%	1.33 (0.84 to 2.04) [p=0.14]			
Tree canopy (ref: 0-9%)				
10-19%		0.87 (0.77 to 1.00) [p=0.025]		
20-29%		0.72 (0.62 to 0.85) [p<0.001]		
≥30%		0.67 (0.57 to 0.80) [p<0.001]		
Grass (ref: 0-4%)				
5-9%			1.07 (0.92 to 1.23) [p=0.19]	
10-19%			1.31 (1.10 to 1.55) [p=0.001]	
20-29%			1.42 (1.16 to 1.71) [p<0.001]	
≥30%			1.47 (1.12 to 1.91) [p=0.002]	
Low-lying vegetation (0-4%)				
5-9%				1.06 (0.96 to 1.16) [p=0.12]
≥10%				0.88 (0.69 to 1.10) [p=0.14]
Age group (ref: 45-54y)				
55-64y	0.96 (0.86 to 1.08) [p=0.26]	0.96 (0.86 to 1.07) [p=0.22]	0.96 (0.86 to 1.08) [p=0.24]	0.96 (0.86 to 1.07) [p=0.23]

© 2019 Astell-Burt T et al. *JAMA Network Open.*

65-74y	1.31 (1.13 to 1.51) [p<0.001]	1.31 (1.13 to 1.50) [p<0.001]	1.31 (1.13 to 1.50) [p<0.001]	1.31 (1.13 to 1.51) [p<0.001]
≥75y	2.50 (2.13 to 2.92) [p<0.001]	2.51 (2.15 to 2.92) [p<0.001]	2.53 (2.16 to 2.94) [p<0.001]	2.50 (2.14 to 2.93) [p<0.001]
Gender (ref: Male)				
Female	0.77 (0.71 to 0.84) [p<0.001]	0.77 (0.71 to 0.84) [p<0.001]	0.77 (0.71 to 0.84) [p<0.001]	0.77 (0.71 to 0.84) [p<0.001]
Annual household income (ref: $0-$19,999)				
$20,000-$29,999	0.89 (0.76 to 1.03) [p=0.06]	0.89 (0.76 to 1.03) [p=0.06]	0.89 (0.76 to 1.02) [p=0.05]	0.89 (0.76 to 1.03) [p=0.06]
$30,000-$39,999	0.78 (0.66 to 0.92) [p=0.001]	0.79 (0.67 to 0.92) [p=0.002]	0.78 (0.66 to 0.92) [p=0.002]	0.78 (0.66 to 0.92) [p=0.001]
$40,000-$49,999	0.78 (0.66 to 0.93) [p=0.002]	0.79 (0.66 to 0.93) [p=0.003]	0.79 (0.66 to 0.93) [p=0.003]	0.79 (0.66 to 0.93) [p=0.002]
$50,000-$69,999	0.65 (0.55 to 0.76) [p<0.001]	0.65 (0.55 to 0.76) [p<0.001]	0.65 (0.55 to 0.77) [p<0.001]	0.65 (0.55 to 0.76) [p<0.001]
$70,000+	0.55 (0.47 to 0.64) [p<0.001]	0.55 (0.47 to 0.64) [p<0.001]	0.55 (0.47 to 0.64) [p<0.001]	0.55 (0.47 to 0.63) [p<0.001]
Not stated	0.83 (0.72 to 0.94) [p=0.002]	0.83 (0.73 to 0.94) [p=0.002]	0.83 (0.73 to 0.94) [p=0.001]	0.83 (0.73 to 0.94) [p=0.001]
Highest educational qualification (ref: None)				
School	0.77 (0.66 to 0.89) [p<0.001]	0.77 (0.66 to 0.90) [p<0.001]	0.77 (0.66 to 0.89) [p<0.001]	0.77 (0.66 to 0.89) [p<0.001]
High school	0.81 (0.68 to 0.97) [p=0.009]	0.82 (0.68 to 0.97) [p=0.013]	0.82 (0.69 to 0.98) [p=0.014]	0.81 (0.68 to 0.97) [p=0.009]
Trade	0.84 (0.70 to 0.99) [p=0.018]	0.84 (0.71 to 0.99) [p=0.019]	0.84 (0.71 to 0.99) [p=0.019]	0.84 (0.70 to 0.99) [p=0.021]
Certificate/Diploma	0.68 (0.58 to 0.80) [p<0.001]	0.69 (0.59 to 0.80) [p<0.001]	0.69 (0.59 to 0.80) [p<0.001]	0.68 (0.58 to 0.80) [p<0.001]
University	0.59 (0.50 to 0.69) [p<0.001]	0.60 (0.51 to 0.70) [p<0.001]	0.60 (0.51 to 0.70) [p<0.001]	0.59 (0.50 to 0.69) [p<0.001]
Not stated	1.05 (0.72 to 1.44) [p=0.43]	1.05 (0.73 to 1.45) [p=0.41]	1.05 (0.72 to 1.44) [p=0.43]	1.04 (0.72 to 1.44) [p=0.43]
Economic status (ref: Employed)				
Retired	1.21 (1.07 to 1.35) [p=0.002]	1.21 (1.07 to 1.36) [p=0.002]	1.21 (1.07 to 1.35) [p=0.001]	1.20 (1.06 to 1.36) [p=0.002]
Unemployed	1.72 (1.26 to 2.28) [p<0.001]	1.73 (1.26 to 2.29) [p<0.001]	1.72 (1.27 to 2.27) [p<0.001]	1.72 (1.25 to 2.28) [p=0.001]
Unpaid work	0.99 (0.68 to 1.37) [p=0.46]	1.00 (0.69 to 1.38) [p=0.48]	1.00 (0.69 to 1.39) [p=0.48]	1.00 (0.69 to 1.38) [p=0.47]
Disabled	2.81 (1.92 to 3.94) [p<0.001]	2.77 (1.89 to 3.87) [p<0.001]	2.81 (1.91 to 3.92) [p<0.001]	2.81 (1.92 to 3.92) [p<0.001]
Homemaker	1.24 (0.98 to 1.54) [p=0.037]	1.25 (0.98 to 1.55) [p=0.034]	1.24 (0.97 to 1.53) [p=0.041]	1.24 (0.97 to 1.53) [p=0.039]
Other (e.g. study)	1.60 (1.16 to 2.13) [p=0.002]	1.61 (1.17 to 2.16) [p=0.002]	1.59 (1.16 to 2.12) [p=0.002]	1.59 (1.16 to 2.11) [p=0.002]

© 2019 Astell-Burt T et al. *JAMA Network Open.*

Couple status (ref: Not in a couple)				
In a couple	0.79 (0.72 to 0.87) [p<0.001]	0.80 (0.73 to 0.88) [p<0.001]	0.78 (0.71 to 0.86) [p<0.001]	0.79 (0.72 to 0.87) [p<0.001]

© 2019 Astell-Burt T et al. *JAMA Network Open.*
